# READY-T1D–assessment of Research and Service Delivery Readiness for paediatric Type 1 Diabetes: a multi-country cross-sectional study

**DOI:** 10.1016/j.eclinm.2026.104067

**Published:** 2026-07-09

**Authors:** John T. Figi, Hugh Pearson, Salma Ahkouk, Bulbul Ahmed, Amos Ankotche, Pablo Aschner, Abdul Basit, Yay Chantana, Khun Leangchhun, Alpha Mamadou Diallo, Sérgio Atala Dib, Dung-Chi Vu, Vani Hebbal Nagarajappa, Ha Nguyen Thu, Zineb Imane, Amadou Kaké, Prasanna Kumar KM, Iv Malene, Ban Manet, Camille M. Mba, Jean Claude Mbanya, Maïmouna Ndour Mbaye, Babacar Niang, Nurain Mohd Noor, Men Panha, Aman Pulungan, Vijay Sheker Reddy Danda, Maria del Pilar Núñez Saavedra, Ana Fernanda Sánchez, Mahamane Sani, Olety Sathyanarayana Santhosh, Segundo Nicolás Seclén Santisteban, Laura Maria Cesar Schiesari, Getahun Tarekegn, Ny Theary, Agustini Utari, Phoebe Wamalwa, Nguon Yaneth, Muhammad Yazid Jalaludin, Bedowra Zabeen, Brooke E. Forde, Maia J. Cullen, Zachary J. Ward, Zia Shakir, Che L. Reddy, Rifat Atun

**Affiliations:** a Health Systems Innovation Lab, Harvard University, Boston, MA, USA; b Department of Global Health and Population, Harvard TH Chan School of Public Health, Harvard University, Boston, MA, USA; c Faculty of Medicine and Pharmacy of Rabat, Mohammed V University, Rabat, Morocco; d Paediatric Diabetes Care & Research Center (PDRC), Bangladesh Institute of Research & Rehabilitation in Diabetes, Endocrine & Metabolic Disorders (BIRDEM), Dhaka, Bangladesh; e Department of Internal Medicine, Endocrinology and Geriatrics, Unit of Training and Research, Medical Science of Abidjan, University of Côte D'Ivoire, Abidjan, Côte d'Ivoire; f Endocrinology Unit, Javeriana University and San Ignacio University Hospital, Bogotá, Colombia; g Indus Diabetes and Endocrinology Center (IDEC), Indus Hospital and Health Network (IHHN), Karachi, Pakistan; h Jayavarman VII Hospital, (Kantha Bopha III, Siem Reap), Cambodia; i Department of Endocrinology and Metabolic Diseases, University Hospital of Donka, Conakry, Guinea; j Department of Medicine, Division of Endocrinology, Universidade Federal de São Paulo, São Paulo, Brazil; k Vietnam National Children's Hospital, Hanoi, Vietnam; l Department of Paediatrics, Indiragandhi Institute of Child Health, Bangalore, India; m Department of Endocrinology Paediatrics, Mohammed V University Medical School, Hôpital d'Enfants, Rabat, Morocco; n Centre for Diabetes and Endocrine care, Bengaluru, India; o Kantha Bopha Children's Hospital, Phnom Penh, Cambodia; p CDiC, Centre National d’Obésité, Hôpital Central, Yaoundé, Cameroon; q Department of Public Health, Faculty of Medicine and Biomedical Sciences, University of Yaoundé 1, Yaoundé, Cameroon; r Abass Ndao Hospital, Dakar, Senegal; s Albert Royer Children's Hospital, Dakar, Senegal; t Malaysian Endocrine and Metabolic Society, Kuala Lumpur, Malaysia; u Cambodia-China Friendship Preah Kossamak Hospital, Cambodia; v Child Health Department, Faculty of Medicine, Universitas Indonesia, Jakarta, Indonesia; w Gandhi Medical College & Hospital, Hyderabad, Telangana, India; x Asociación Colombiana de Diabetes, Bogotá, Colombia; y Casa de la Diabetes, Cuenca, Ecuador; z Department of Endocrinology Diabetology Nutrition, Hôpital Général de Référence (General Reference Hospital), Niamey, Niger; aa Karnataka Institute of Endocrinology and Research, Old Madras Road, Indiranagar post, Bengaluru, Karnataka, India; ab Universidad Peruana Cayetano Heredia & Juvenil Diabetes Association, Lima, Peru; ac São Paulo School of Business Administration, Fundação Getulio Vargas, São Paulo, Brazil; ad Ethiopian Diabetes Association, Addis Ababa, Ethiopia; ae Department of Pediatrics, Faculty of Medicine, Universitas Diponegoro, Semarang, Indonesia; af Department of Pediatrics, Kajiado County Referral Hospital, Nairobi, Kenya; ag Department of Paediatrics, Faculty of Medicine, Universiti Malaya, Malaysia; ah Changing Diabetes in Children Programme, Diabetic Association of Bangladesh, Dhaka, Bangladesh; ai Center for Health Decision Science, Harvard TH Chan School of Public Health, Harvard University, Boston, MA, USA; aj Department of Health Policy and Management, Harvard TH Chan School of Public Health, Harvard University, Boston, MA, USA; ak Department of Global Health and Social Medicine, Harvard Medical School, Boston, MA, USA

**Keywords:** Clinic assessment, Type 1 diabetes, Health systems, Global health, Research preparedness

## Abstract

**Background:**

Type 1 diabetes (T1D) incidence is rising globally, particularly in low- and middle-income countries (LMICs) that face major gaps in access and treatment and high levels of preventable mortality. We developed READY-T1D (Research and Service Delivery Readiness in Type 1 Diabetes) to assess service provision and research preparedness in clinics providing T1D services to children and adolescents as part of the Global Collaborative for Changing Diabetes in Children (GC-CDiC).

**Methods:**

From November 15th, 2024, to November 14th, 2025, we conducted a cross-sectional online survey of clinics caring for individuals living with T1D aged ≤20 years across 21 countries participating in GC-CDiC. The tool enabled assessment and scoring in two domains, Service Provision and Research Preparedness, each made up of five components (score on 0–4 scale, where 4 = highest readiness). Descriptive statistics were generated for clinic characteristics, item-level metrics, component scores, and domain scores, and compared across countries.

**Findings:**

We analysed 244 clinics (69% CDiC centres, 14% satellites, 17% non-CDiC) across 21 countries. Mean scores were 2.5 (SD 0.6) for Service Provision and 1.8 (SD 0.8) for Research Preparedness, with substantial variation within countries. While resource management and accessibility scored highly, testing facilities and staffing were weaker. Basic care (HbA1c, complication screening) was common, but advanced diagnostics and continuous glucose monitoring were rare. In research, clinics reported high scheduled visit frequency, but often lacked secure data systems, funding, and ethical governance.

**Interpretation:**

Clinics providing care for children and adolescents with T1D in GC-CDiC participating countries possess core care elements and report frequent scheduled patient contact but lack comprehensive readiness for service delivery and research. Capacity varies widely both within and between countries. Priorities for investment for capacity development to improve care in studied clinics include multidisciplinary staffing, digital infrastructure, and research governance to support the Global T1D Cohort Study.

**Funding:**

The study was supported by an unrestricted grant from 10.13039/501100004191Novo Nordisk, which had no role in study design, protocol development, data collection, analysis, interpretation, or writing of the report.

**Research in context**

**Evidence before this study**

We searched PubMed for articles published from Jan 1, 2000, to Feb 1, 2026, using combinations of the terms “type 1 diabetes”, “children”, “adolescents”, “service readiness”, “health system readiness”, “capacity assessment”, “clinic capacity”, “research readiness”, and “research preparedness”. We also reviewed WHO reports and existing service readiness tools (e.g., the WHO Service Availability and Readiness Assessment), as well as publications related to global type 1 diabetes (T1D) epidemiology. We found substantial literature documenting poor access to insulin, monitoring supplies, specialist care, and outcomes for children and adolescents with T1D in low-resource settings. However, we did not identify any standardised, quantitative tools specifically designed to assess clinic-level readiness for both service provision and research in paediatric and adolescent T1D, nor any multi-country assessments using a common framework.

**Added value of this study**

This study introduces READY-T1D (Research and Service Delivery Readiness in Type 1 Diabetes), a structured tool developed to assess both service provision and research preparedness for paediatric and adolescent T1D clinics in low- and middle-income countries (LMICs). Applying READY-T1D to 244 clinics across 21 GC-CDiC countries, we provide standardised, directly comparable data on clinic readiness in this context, which to our knowledge has not previously been assessed using a common quantitative framework across this range of settings. We show that while most clinics possess core elements of T1D care (e.g., HbA1c testing, routine clinical and complication screening), they frequently lack comprehensive readiness for advanced care and research participation. We identify large, quantifiable gaps in multidisciplinary staffing, advanced diagnostics and technologies, digital infrastructure, secure data systems, and governance structures. We also document marked within-country heterogeneity, demonstrating that readiness cannot be inferred from country income level alone. Beyond description, READY-T1D translates these findings into a practical framework for targeting capacity-building investments and has already been used to inform co-designed training programmes and workshops across multiple regions.

**Implications of all the available evidence**

Taken together with prior evidence on global T1D burden and access gaps, our findings indicate that many paediatric and adolescent diabetes clinics in GC-CDiC participating countries report characteristics, such as frequent scheduled follow-up, that may support engagement in longitudinal care and research, but are constrained by specific deficits in infrastructure, governance, and workforce capacity. For initiatives such as the Global T1D Cohort Study, this means that investments should prioritise (1) strengthening multidisciplinary teams, including specialist nurses, dietitians, psychologists, and social workers; (2) digital transformation, including interoperable and secure data systems; and (3) robust data governance and research administration. The pronounced variation within countries supports a hub-and-spoke or tiered implementation model, in which better-resourced clinics mentor and support lower-readiness sites through phased capacity-building. The approach used in READY-T1D may provide a starting point for assessing readiness in other settings or conditions, though further adaption and validation would be required before application beyond the current context. Systematic, clinic-level readiness assessment represents a valuable next step in designing global cohort studies and implementation efforts aiming to reduce inequities in T1D outcomes for children and adolescents worldwide.

## Introduction

Type 1 diabetes (T1D) incidence is rising worldwide, particularly in low- and middle-income countries (LMICs) which experience the largest gaps in access to care and treatment and the highest mortality rates globally.^1^^,^^2^ Health systems in these settings frequently lack trained multidisciplinary teams, reliable access to insulin and monitoring supplies, and basic diagnostic services.^3^ Furthermore, digital systems for data management are often weak or absent, resulting in fragmented care and the under-representation of children from LMICs in global research.^4^

Since 2009, the Changing Diabetes in Children (CDiC) partnership led by Novo Nordisk has addressed these barriers by establishing a network of centres and satellite clinics that provide free insulin, monitoring supplies, and structured education to over 60,000 children in 30 countries.^5^ The Global Collaborative for Changing Diabetes in Children (GC-CDiC) was established in 2022 to advance the understanding and management of T1D in children and adolescents across these countries and globally (GC-CDiC is not limited to only CDiC sites or countries). GC-CDiC seeks to extend this infrastructure to support initiatives aimed at improving early diagnosis, appropriate care, and better outcomes for children and adolescents living with T1D, including the Global T1D Cohort Study.^6^ However, supporting such a diverse network requires a standardised understanding of existing clinic capacity. Currently, no common tool exists to characterise service provision and research readiness in paediatric and adolescent T1D clinics globally.

To address this gap, we developed READY-T1D (Research and Service Delivery Readiness in Type 1 Diabetes), a structured assessment tool designed to capture clinic-level capacity. We deployed the READY-T1D survey across 21 LMICs to quantify service provision and research preparedness. By establishing comparable benchmarks, we aim to identify critical infrastructure gaps, guide targeted capacity-strengthening, and support the equitable inclusion of LMIC clinics in Global T1D research.

## Methods

### Study design and participants

From November 15th, 2024 to November 14th, 2025 we conducted a cross-sectional survey of clinics providing care for children and adolescents (≤20 years) with T1D in countries currently participating in GC-CDiC including: Bangladesh, Brazil, Cambodia, Cameroon, Colombia, Côte d’Ivoire, Ecuador, Ethiopia, Ghana, Guinea, India, Indonesia, Kenya, Malawi, Malaysia, Morocco, Nigeria, Pakistan, Peru, Senegal, and Vietnam. Clinics were eligible if they provided ongoing clinical care to paediatric and adolescent patients with T1D, regardless of level of care (primary, secondary, or tertiary), sector (public, private, or mixed), or CDiC affiliation.

### Recruitment

We distributed the survey through existing GC-CDiC networks and national paediatric and adolescent diabetes communities in the 21 countries involved in the study. Country leads identified eligible clinics and emailed survey links to senior clinicians or administrators capable of reporting on both clinical practice and organisational structure. Leads monitored completion via summary reports to target follow-up and clarify questions. Participation was voluntary.

### READY-T1D development

We developed READY-T1D to quantify clinic-level readiness for service delivery and research in LMICs, adapting the WHO Service Availability and Readiness Assessment^7^ and Atun's health-systems framework.^8^ The tool comprises a descriptive module (clinic characteristics) and two scored domains, each divided into five components.

#### Service provision

Assesses Clinical Expertise and Staffing (multidisciplinary mix, provider-to-patient ratios); Health Services (screening, insulin and glucose monitoring methods, DKA management, education); Resource Management (digital infrastructure, stock reliability); Testing Facilities (lab capacity from HbA1c to genetic testing); and Accessibility (language support and travel time).

#### Research preparedness

Assesses Data Management (staffing, integration, security); Patient Engagement (follow-up frequency—efforts put in by the clinic to engage with patients); Research Capabilities (Good Clinical Practice (GCP) training, data privacy, ethics access, quality assuarance procedures); Finance and Administration (funding history, protected research time); and Collaboration (participation in national or international networks).

The full READY-T1D questionnaire and scoring rules are provided in Supplementary Table S1.

### Scoring and refinement

Metrics derived from survey items were mapped to a 0–4 readiness scale, where 0 indicates the lowest and 4 the highest level of readiness. Scoring used conditional rules to avoid penalising clinics for services outside their scope (e.g., inpatient DKA management in outpatient-only sites).

The tool was refined following a pilot study of 146 clinics in five countries, which informed the expansion of the scoring scale (from 0–2 to 0–4), the clarification of phrasing (including reducing ambiguity in phrasing of questions and responses to ensure response reproducibility), and the addition of specific items on complications, DKA, and governance. Pilot study methodology and results are provided in Supplementary File S1.

### Translation

The survey was administered in English, French, Spanish, and Portuguese via Qualtrics. The French version was developed by a professional translation service specialising in Francophone African contexts, with back-translation by a bilingual native Senegalese French/Canadian English speaker. The Spanish version was translated by a native South American Spanish/fluent English speaker and back-translated by a native English/fluent Spanish speaker. The Portuguese version was translated by a native South American Portuguese/fluent English speaker. Where direct translation was not possible, culturally equivalent wording was used to ensure clarity.

### Data analysis

We cleaned data in Microsoft Excel (version 16.0.1) to assess completeness and consistency. Surveys with less than 100% completion were excluded (with completion of clinic practitioner types but not numbers of each type being counted as complete). We calculated descriptive statistics (means, SDs, counts, percentages) for clinic characteristics, item-level metrics, and domain scores. Service provision and research preparedness scores were compared descriptively across countries.

### Ethics

The study protocol was reviewed by the Harvard Longwood Campus Institutional Review Board (IRB) and determined to not constitute human subjects research (Protocol IRB25-1007). Respondents provided electronic informed consent prior to participation. No individual patient identifiers were collected.

### Role of the funding source

The funder had no role in study design, protocol development, data collection, data analysis, data interpretation, or writing of this report.

## Results

### Participating clinics

We received 408 responses from which 244 complete responses were analysed from 21 countries. The majority of the 164 incomplete responses contained only metadata due to the survey link being opened but not started (n = 151/164, 92%), of which 132/151 (87%) could be linked to surveys completed later by matching IP addresses within the metadata. Overall, 12/164 (7%) unique incomplete surveys could not be matched to a survey later completed in full. Full details on incomplete surveys are included in Supplementary File S2. The response rate for completed surveys was 85% (244/287), with country response rates included in Supplementary Table S2.

Country participation was concentrated in a small number of settings: the largest contributions came from India (n = 51), Malaysia (n = 33), Ethiopia (n = 32), Kenya (n = 28), and Pakistan (n = 24). The majority of clinics were CDiC centres (69%, n = 168), followed by CDiC satellite centres (14%, n = 35), and non-CDiC clinics (17%, n = 41). Ownership was predominantly public (73%, n = 178), with 23% (n = 57) private, and 4% (n = 9) mixed facilities.

Service scope was heterogenous, 72% (n = 176) of clinics provided both inpatient and outpatient care, while 27% (n = 67) were outpatient-only, and <1% (n = 1) clinic was inpatient only. Facility types included teaching or academic hospitals (21%, n = 51), primary care sites (19%, n = 46), speciality outpatient centres (17%, n = 42), district or regional hospitals (16%, n = 38), tertiary hospitals (14%, n = 33), general hospitals (13%, n = 32), and community health centres (1%, n = 2). The mean number of registered children with T1D per clinic was 165 (SD 286), with a median of 62.

### Overall readiness scores

**Fig. 1:**
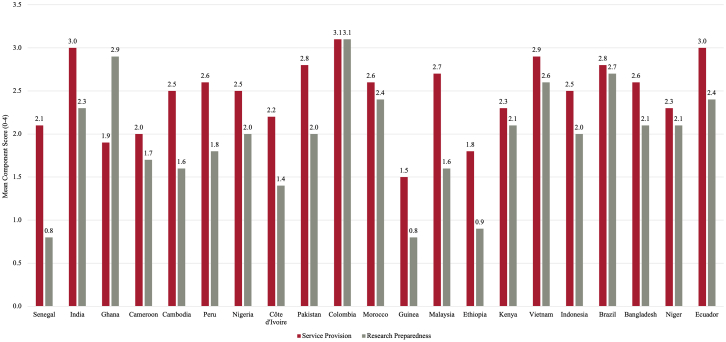
**Country Mean Scores: Service Provision and Research Preparedness.** The y-axis represents mean READY-T1D scores on a 0–4 scale, where 0 indicates the lowest level of readiness and 4 indicates the highest.

**Fig. 2:**
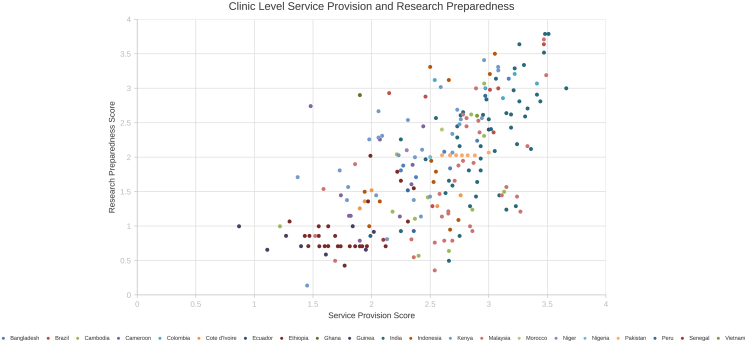
**Clinic-level service provision and research preparedness scores**.

The mean score across all clinics was 2.5 (SD 0.6) for Service Provision and 1.8 (SD 0.8) for Research Preparedness. Eighteen percent of clinics had Service Provision scores ≥3.0, and 11% had Research Preparedness ≥3.0. Scores for both domains showed substantial variation between and within countries, as illustrated in Fig. 1 (Country Mean Scores) and Fig. 2 (Clinic Mean Scores). Detailed item-level metrics are summarised in Table 1, and country-level breakdowns are provided in Table 2.

**Table 1:** Question Summary Data (n = 244 clinics).

Clinical Expertise	Component	n Clinics	% Total
Q1: Please indicate which of the below practitioners are available at your centre to treat T1D in patients ≤20 years old and how many	Nurses	148	61
	Endocrinologists/Diabetologists	151	62
	Dietician	165	68
	General physician/non-specialist	124	51
	Paediatrician (non-endo)	124	51
	Specialist nurses	110	45
	Psychologist/counsellor	99	41
	Medical assistants	97	40
	Paediatric endocrinologist	86	35
	Social worker	83	34
	Trainee physician	64	26
**Health services**			
Q2: Please indicate which services to screen for T1D complications are routinely provided	Clinical Exam	241	99
	Eye Fundus Exam	178	73
	Foot Exam	196	80
	Height and weight measurement	237	97
Q3: What equipment do most (>50%) children use to check blood glucose?	Glucometer + test strips + syringes	233	95
	Continuous Glucose Monitoring Device	11	5
	None	0	0
Q4: What equipment do you PROVIDE for blood glucose/ketone monitoring (free or paid)?	For Free: Glucometer	190	78
	For Free: Test strips	190	78
	For Free: Urine ketone strips	32	13
	For Free: Capillary ketone monitor	14	6
	For Free: CGM	23	9
	Paid: Glucometer	59	24
	Paid: Test strips	58	24
	Paid: CGM	58	24
	Paid: Capillary Ketone Monitor	25	10
	Paid: Urine Ketone Strips	52	21
	None	14	6
Q5: What insulin delivery do most (>50%) children use?	Subcutaneous Insulin Syringe	80	33
	Subcutaneous Insulin Pen	163	67
	Insulin Pump	1	<1
			
Q6: Is acute DKA managed at your facility? (from diagnosis to discharge)	No	15	6
	Only diagnosis	53	22
	Yes - From diagnosis to treatment to discharge	176	72
Q7: Which of the following does your clinic have to treat acute DKA? (% calculated from clinics not answering ‘No’ to prior question, n = 229)			
	Capillary glucose monitor	195	85
	Capillary ketone monitor	85	37
	POC blood gas analysis	104	45
	Continuous cardiac monitoring/pulse oximetry	152	66
	Fast-acting insulin	215	94
	IV fluids	215	94
	Potassium replacement	176	77
Q8: What proportion receive structured diabetes education at diagnosis?	0–25%	29	12
	25–50%	32	13
	50–75%	38	16
	75–100%	145	59
Q9: What proportion receive these services without paying at point of care?	None	16	7
	<25%	23	10
	25–50%	37	15
	50–75%	40	16
	75–<100%	40	16
	100%	88	36
Q10: Are there private spaces for patient consultations?	Yes	189	77
	No	55	23
			
Q11: Is telehealth capability available?	Yes	119	49
	No	125	51
**Resource management**			
Q12: What digital devices are in place to manage patient data?	Both computers and mobile devices	109	45
	Either computers or mobile devices	81	33
	No computer or mobile device	54	22
Q13: Are software programs available and up to date for digital patient information?	Software available and up to date	92	38
	Software available but not up to date	55	23
	No software available	97	40
Q14: How often does your facility experience insulin stock outs?	Stock outs 0–5% (0–19 days)	140	57
	Stock outs 5–10% (20–40 days)	50	20
	Stock outs 10–15% (41–60 days)	14	6
	Stock outs 15–20% (61–80 days)	7	3
	Stock outs 20–30% (81–120 days)	13	5
	Stock outs >30% of year (>120 days)	11	5
	Insulin not stocked/provided	9	4
Q15: How often does your facility experience glucose equipment stock outs?	Stock outs 0–5% (0–19 days)	121	50
	Stock outs 5–10% (20–40 days)	38	16
	Stock outs 10–15% (41–60 days)	17	7
	Stock outs 15–20% (61–80 days)	6	2
	Stock outs 20–30% (81–120 days)	13	5
	Stock outs >30% of year (>120 days)	26	11
	Equipment not stocked/provided	23	9
Q16: Does your facility have software for inventory management?	Software-based inventory management	48	20
	Manual inventory management	155	64
	No inventory management	41	17
Q17: Is the clinic equipped with stable internet connectivity?	Internet >75% of time	122	50
	Internet 50–75% of time	26	11
	Intermittent internet	42	17
	No internet	54	22
**Testing facilities**			
Q18: Which lab tests are available for T1D patients?	HbA1c	240	98
	Lipid profile	172	70
	Serum creatinine	185	76
	Urine creatinine	122	50
	Thyroid function test	140	57
	Full blood count	186	76
	None	2	1
Q19: Which tests are available/accessed?	Oral glucose tolerance testing	143	59
	Pancreatic autoantibody	86	35
	C-peptide testing	112	46
Q20: Does the facility have access to genetic testing for T1D patients?	Yes	39	16
	No	205	84
**Accessibility**			
Q21: What proportion of T1D patients travel over 60 min to reach your centre?	66–100%	54	23
	33–66%	142	58
	<33%	43	18
	None	5	2
Q22: Is the clinic equipped for language support for diverse populations?	Support for multiple languages	146	60
	Support for one local language	42	17
	Not equipped	56	23
**Data management**			
Q23: Is the clinic adequately staffed for patient data recording for research?	>75% of required staff	81	33
	25–75% of required staff	88	36
	<25% of required staff	75	31
Q24: Can systems integrate electronic patient records from external health centres?	Full integration (75–100%)	45	18
	Majority integration (25–75%)	22	9
	Some integration (<25%)	20	8
	No integration (0%)	76	31
	No digital systems	81	33
Q25: Does the clinic have ability to store study data securely?	>75% securely stored	112	46
	50–75% securely stored	16	7
	<50% securely stored	28	11
	Ability but done 0%	20	8
	No ability	68	28
**Patient engagement**			
Q26: Does the clinic conduct regular follow-up with T1D patients?	≥4 times/year (every 3 months or more)	186	76
	3 times/year (every 4 months)	37	15
	2 times/year (every 6 months)	10	4
	Once a year	7	3
	No regular follow-up	4	2
**Research capabilities**			
Q27: What percentage of research staff have GCP certification?	>75% staff certified	48	20
	50–75% staff certified	48	20
	25–50% staff certified	36	15
	<25% staff certified	112	46
Q28: Do staff have experience in clinical research studies?	>75% staff experienced	35	14
	50–75% staff experienced	28	11
	25–50% staff experienced	47	19
	<25% staff experienced	134	55
Q29: Are there established guidelines for handling digital patient data?	Guidelines exist, followed >75%	69	28
	Guidelines exist, followed 50–75%	48	20
	Guidelines exist, followed <50%	30	12
	No guidelines	97	40
Q30: Are staff trained in data privacy in digital environment?	>75% staff trained	90	37
	50–75% staff trained	24	10
	25–50% staff trained	19	8
	<25% staff trained	41	17
	Not trained	70	29
Q31: Do you have an IRB/Ethics Committee locally?^a^	Local IRB	89	36
	Affiliate IRB	53	22
	No IRB available	102	42
Q32: How frequently does your IRB/Ethics Committee meet?^a^	Weekly	0	0
	Monthly	52	21
	Every 2–3 months	39	16
	Less frequently than every 3 months	41	17
	No IRB	112	46
Q33: Are there quality assurance procedures for data collection?	Procedures exist and followed	101	41
	Procedures exist but not followed	37	15
	No procedures	106	43
**Finance and administration**			
Q34: Have you previously acquired grants/funding for research?	Yes	71	29
	No	173	71
Q35: How much time can admin staff allocate to research support (avg hours per staff)?	N/A	5	2
	<4 h/week per staff	122	50
	4–8 h/week per staff	62	25
	8–12 h/week per staff	21	9
	12–16 h/week per staff	8	3
	>16 h/week per staff	26	11
**Collaboration and networking**			
Q36: Does the clinic participate in diabetes research networks?	National + international networks	65	27
	National networks only	84	34
	Does not participate	95	39

a Q31 and Q32—18 sites reported incongruent IRB/Ethics Committee access, with 13 sites reporting affiliate access in Q31 but no access in Q32, 1 site reporting local access in Q31 but no access in Q32, and 4 sites reporting no access in Q31 but access in Q32. The largest group of these, those reporting access in Q31 but no access in Q32 (n = 14), likely represents respondents that did have affiliate or local IRB/Ethics Committee access, but did not know how frequently they met.

**Table 2:** READY-T1D scores by country.

Country	IND	BGD	BRA	KHM	CMR	COL	CIV	ECU	ETH	GHA	GIN	IDN	KEN	MYS	MAR	NER	NGA	PAK	PER	SEN	VNM	Mean
n	51	7	7	12	10	5	5	1	32	1	8	12	28	33	1	1	1	24	3	1	1	244
Clinical expertise and staffing																						
Q1: Please indicate which of the below practitioners are available at your centre to treat T1D in patients ≤20 years old and how many	2.1	2.7	2.6	1.2	2.6	2.2	1.8	2.2	1.1	2.2	1.1	2.1	2.4	2.0	1.8	4.0	1.5	1.2	2.7	2.2	1.1	1.9
Adequacy of staffing relative to T1D caseload score	1.5	3.9	0.7	1.3	2.8	1.4	2.4	4.0	3.8	4.0	0.0	1.0	1.4	2.8	4.0	4.0	0.0	3.3	4.0	4.0	3.0	2.3
Health services																						
Q2: Please indicate which services to screen for T1D complications are routinely provided	3.7	2.3	4.0	3.0	2.8	2.8	3.4	4.0	3.7	4.0	2.8	3.3	3.4	3.6	3.0	3.0	2.0	4.0	3.7	4.0	3.0	3.5
Q3: What equipment do most (>50%) children use to check blood glucose?	2.1	2.0	2.3	2.7	2.0	3.2	2.0	2.0	2.0	2.0	2.0	2.0	2.0	2.1	2.0	2.0	2.0	2.0	2.0	2.0	2.0	2.1
Q4: What equipment do you PROVIDE for blood glucose/ketone monitoring (free or paid)?	2.5	3.4	2.1	2.5	2.6	2.8	2.7	2.4	1.6	2.4	2.1	2.2	2.1	2.7	4.0	2.4	2.4	1.6	1.3	4.0	N/A	2.2
Q5: What insulin delivery do most (>50%) children use?	1.7	0.0	2.0	1.5	0.2	2.0	0.0	2.0	0.0	0.0	0.0	2.0	2.0	1.9	2.0	0.0	2.0	2.0	1.3	0.0	0.0	1.4
Q7: Which of the following does your clinic have to treat acute DKA?	3.7	2.0	4.0	3.2	3.5	4.0	3.2	N/A	3.9	4.0	2.0	4.0	3.2	3.6	4.0	4.0	4.0	2.0	1.3	4.0	4.0	3.5
Q8: What proportion receive structured diabetes education at diagnosis?	3.5	3.3	2.9	2.9	2.8	3.5	3.7	4.0	0.5	4.0	3.5	3.3	3.4	3.0	4.0	2.7	4.0	4.0	1.8	4.0	4.0	3.0
Q9: What proportion receive services without paying at point of care?	2.5	3.1	4.0	2.9	3.0	2.2	4.0	1.6	1.9	3.2	3.0	3.3	3.0	3.0	2.4	0.8	N/A	4.0	3.7	N/A	2.4	2.9
Q10: Are there private spaces for patient consultations?	3.8	4.0	2.3	3.3	2.4	4.0	2.4	4.0	1.4	0.0	3.0	3.0	3.1	3.2	4.0	4.0	4.0	4.0	2.7	0.0	4.0	3.1
Q11: Is telehealth capability available?	3.2	1.7	3.4	2.3	0.4	4.0	0.8	4.0	0.1	0.0	0.0	2.0	1.0	1.2	4.0	4.0	0.0	4.0	4.0	4.0	0.0	2.0
Resource management																						
Q12: What digital devices are in place to manage patient data?	3.8	3.4	4.0	3.7	2.8	4.0	4.0	4.0	0.6	4.0	0.0	4.0	3.3	3.4	4.0	4.0	4.0	4.0	2.7	0.0	4.0	3.1
Q13: Are software programs available and up to date for digital patient information?	3.0	1.4	3.1	2.2	0.8	2.8	1.6	2.0	0.3	2.0	0.5	1.3	2.0	1.2	4.0	0.0	2.0	4.0	1.3	2.0	4.0	2.0
Q14: How often does your facility experience insulin stock outs?	3.5	4.0	2.9	3.1	3.3	3.6	2.7	3.2	3.1	0.0	4.0	2.6	3.1	3.1	0.8	4.0	4.0	4.0	4.0	3.2	4.0	3.3
Q15: How often does your facility experience glucose equipment stock outs?	3.4	4.0	3.8	3.1	2.1	3.8	2.6	4.0	2.9	0.0	1.0	2.1	1.9	3.4	0.0	N/A	N/A	4.0	4.0	2.4	N/A	3.0
Q16: Does your facility have software for inventory management?	2.9	2.3	3.1	3.3	2.8	3.6	2.4	4.0	2.0	2.0	3.3	3.7	2.6	2.9	2.0	2.0	2.0	2.0	3.3	2.0	4.0	2.7
Q17: Is the clinic equipped with stable internet connectivity?	3.9	3.1	3.6	3.6	0.9	4.0	1.3	4.0	0.9	0.0	0.3	3.3	2.3	2.8	4.0	1.3	2.7	1.3	3.6	2.7	2.7	2.5
Testing facilities																						
Q18: Which lab tests are available for T1D patients?	3.7	3.4	4.0	3.2	1.4	4.0	2.7	0.7	2.5	0.7	2.2	3.6	2.6	3.8	0.7	N/A	3.3	0.7	3.3	0.7	4.0	2.9
Q19: Which tests are available/accessed?	3.3	1.9	3.4	2.3	2.3	3.5	2.0	N/A	1.3	N/A	1.3	1.8	1.7	3.7	2.7	N/A	2.7	4.0	1.8	1.3	4.0	2.8
Q20: Does the facility have access to genetic testing for T1D patients?	1.9	0.0	0.6	0.3	0.8	0.8	0.0	0.0	0.1	0.0	0.0	0.3	0.0	0.9	0.0	0.0	0.0	0.0	0.0	0.0	4.0	0.6
Accessibility																						
Q22: Is the clinic equipped for language support for diverse populations?	3.6	2.6	0.9	2.0	0.6	2.4	1.6	4.0	3.4	4.0	0.3	1.5	2.6	2.9	2.0	0.0	4.0	4.0	1.3	0.0	0.0	2.7
Data management and integration																						
Q23: Is the clinic adequately staffed for patient data recording for research?	2.8	1.4	2.9	1.5	0.4	2.4	0.8	4.0	3.6	0.0	0.3	1.3	2.0	1.0	0.0	0.0	4.0	2.1	1.3	0.0	4.0	2.1
Q24: Can systems integrate electronic patient records from external health centres?	1.7	1.7	2.6	1.9	0.7	2.0	0.2	0.0	0.1	1.0	0.4	2.1	1.3	1.0	2.0	0.0	0.0	4.0	0.7	1.0	0.0	1.5
Q25: Does the clinic have ability to store study data securely?	3.0	2.4	2.7	1.8	1.6	4.0	3.2	4.0	0.4	2.0	1.0	2.7	2.9	1.6	4.0	2.0	1.0	4.0	2.3	1.0	4.0	2.3
Patient engagement																						
Q26: Does the clinic conduct regular follow-up with T1D patients?	3.6	3.0	3.1	3.8	3.7	3.2	3.8	2.0	4.0	4.0	4.0	2.8	3.5	3.6	3.0	4.0	4.0	4.0	3.3	1.0	4.0	3.6
Research capabilities																						
Q27: What percentage of research staff have GCP certification?	1.5	2.7	2.5	0.8	1.9	4.0	0.0	0.0	0.3	2.7	0.5	1.8	1.9	1.5	0.0	1.3	0.0	2.6	0.9	0.0	4.0	1.5
Q28: Do staff have experience in clinical research studies?	1.5	2.1	2.7	1.0	1.5	3.7	0.8	4.0	0.0	4.0	0.0	1.5	1.5	1.0	1.3	4.0	4.0	0.0	1.3	0.0	2.7	1.1
Q29: Are there established guidelines for handling digital patient data?	2.3	2.5	3.4	1.2	1.1	4.0	1.1	2.7	0.2	4.0	1.0	1.4	2.6	1.2	4.0	4.0	0.0	2.7	2.7	0.0	4.0	1.8
Q30: Are staff trained in data privacy in digital environment?	2.4	3.0	3.4	1.3	2.3	4.0	2.2	4.0	0.1	3.0	1.3	1.7	2.4	1.6	4.0	2.0	0.0	4.0	2.3	0.0	4.0	2.1
Q31: Do you have an IRB/Ethics Committee locally?	3.0	2.3	3.1	2.2	2.8	3.2	3.2	4.0	2.4	4.0	2.0	3.8	2.7	2.9	4.0	2.0	4.0	2.0	3.3	2.0	2.0	2.7
Q32: How frequently does your IRB/Ethics Committee meet?	1.7	0.1	2.4	0.5	1.5	2.2	1.4	3.0	0.6	2.0	0.1	1.8	1.2	1.2	1.0	3.0	3.0	0.0	1.3	0.0	3.0	1.1
Q33: Are there quality assurance procedures for data collection?	2.6	4.0	3.1	1.7	2.2	4.0	0.0	2.0	0.6	4.0	0.0	2.7	2.6	2.3	2.0	4.0	4.0	0.0	2.7	2.0	4.0	2.0
Finance and administrative support																						
Q34: Have you previously acquired grants/funding for research?	2.0	1.1	2.3	1.0	1.2	2.4	1.6	0.0	0.4	4.0	0.0	1.0	1.0	1.5	0.0	0.0	0.0	0.0	2.7	0.0	0.0	1.2
Q35: How much time can admin staff allocate to research support (avg hours per staff)?	1.8	0.7	0.7	1.2	0.7	0.4	0.2	2.0	0.0	2.0	0.1	1.8	1.4	0.6	4.0	0.0	0.0	1.0	0.0	0.0	0.0	1.0
Collaboration and networking																						
Q36: Does the clinic participate in diabetes research networks?	2.1	2.3	3.1	1.8	1.8	3.2	0.8	2.0	0.2	4.0	0.5	2.2	2.3	1.6	4.0	4.0	4.0	2.0	0.0	4.0	0.0	1.8
Mean Country Score	2.7	2.4	2.8	2.1	1.9	3.1	1.9	2.7	1.4	2.3	1.2	2.3	2.2	2.3	2.5	2.3	2.3	2.5	2.2	1.6	2.7	
Standard Deviation	*0.5*	*0.4*	*0.5*	*0.5*	*0.3*	*0.2*	*0.3*	*N/A*	*0.3*	*N/A*	*0.3*	*0.6*	*0.5*	*0.6*	*N/A*	*N/A*	*N/A*	*0.1*	*0.6*	*N/A*	*N/A*	

### Service provision

**Fig. 3:**
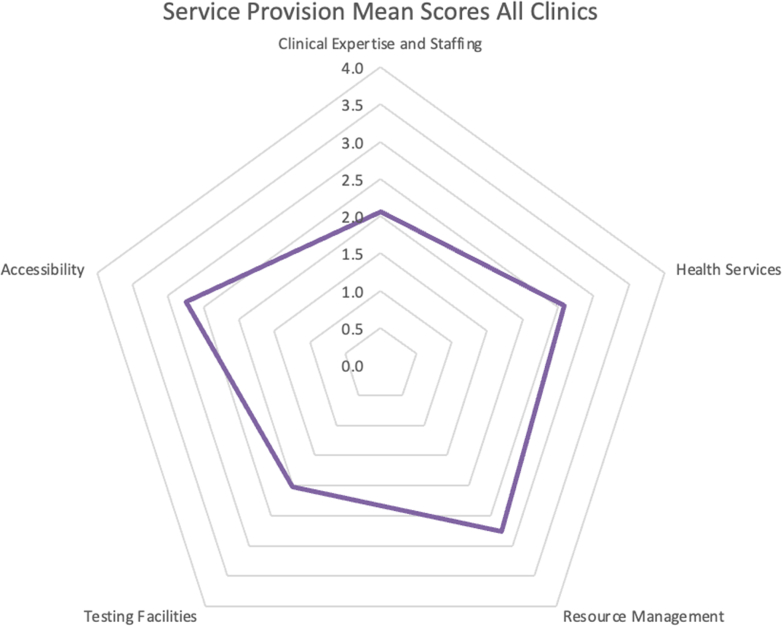
**Mean service provision scores**.

Mean scores for the five service provision components were: Resource Management (2.8, SD 0.9), Accessibility (2.7, SD 1.7), Health Services (2.6, SD 0.6), Clinical Expertise and Staffing (2.1, SD 1.1), and Testing Facilities (1.9, SD 1.1) as shown in Fig. 3.

### Clinical expertise and staffing

75% (n = 183) of clinics reported having an endocrinologist or diabetologist (either paediatric or non-paediatric specialist), while 86% (n = 209) reported at least one nurse (specialist and/or non-specialist). 35% (n = 85) reported having at least one paediatric endocrinologist/diabetologist, 61% (n = 148) reported at least one non-specialist nurse, and 45% (n = 110) reported specialist nurses. Dietitians were present in 68% (n = 165) of clinics, psychologists or counsellors in 41% (n = 99), and social workers in 34% (n = 83). Regarding provider-to-patient ratios, data were available for 62% (n = 152) of clinics. Among these, 69% (n = 105) reported a favourable ratio of <25 children per full-time equivalent (FTE) clinical provider (doctors and nurses), while only 8% (n = 12) reported a ratio of >100 children per FTE provider. However, 38% (n = 92) of clinics did not provide sufficient data on staffing numbers to calculate these ratios, and the caseload of other medical conditions treated at each centre is not reflected in these ratios.

### Health services

For routine screening, 99% (n = 241) of clinics performed clinical examinations, 97% (n = 237) measured height and weight, 80% (n = 196) performed foot examinations, and 73% (n = 178) performed eye fundus examinations. The predominant blood glucose monitoring method was via glucometer with test strips in 95% (n = 233) of clinics, while 5% (n = 11) used continuous glucose monitors (CGM). Regarding affordability, 78% (n = 190) of clinics provided glucometers free of charge, compared to 13% (n = 32) for urine ketone strips, and 9% (n = 23) for CGM. For acute care, 72% (n = 176) of clinics managed diabetic ketoacidosis (DKA) from diagnosis to discharge, 22% (n = 53) provided diagnosis only with onward transfer, and 6% (n = 15) provided no DKA management.

### Resource management

Seventy-eight percent of clinics (n = 190) possessed at least one digital device for patient data, but 22% (n = 54) reported no digital devices. Up-to-date clinical software was reported by 38% (n = 92) of clinics, while 40% (n = 97) had no software for patient information. Regarding supply reliability, 10% (n = 24) of clinics stocking insulin reported stock-outs on >20% of days in the previous year. Internet connectivity was reported as stable (>75% of the time) by 50% (n = 122) of clinics, while 22% (n = 54) reported no internet access.

### Testing facilities

HbA1c testing was available in 98% (n = 240) of clinics. Availability of other tests included: full blood count 76% (n = 186), serum creatinine 76% (n = 185) lipid profiles 70%, oral glucose tolerance testing 59% (n = 143), thyroid function tests 57% (n = 140), C-peptide 46% (n = 112), pancreatic autoantibodies 35% (n = 86), and genetic testing 16% (n = 39).

### Accessibility

In 22% (n = 54) of clinics, an estimated 66–100% of patients travelled more than 60 min to reach the facility, and in a further 58% (n = 142) of clinics, 33–66% of patients faced journeys of this length. Only 2% (n = 5) of clinics reported that no patients travelled more than an hour. Regarding language support, 60% (n = 146) of clinics provided care in multiple languages and 17% (n = 42) in a single local language, while 23% (n = 56) reported no structured language support.

### Research preparedness

**Fig. 4:**
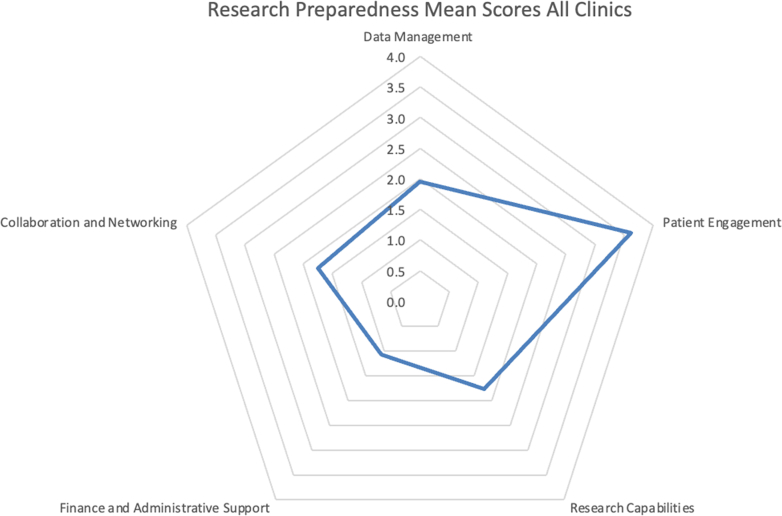
**Mean research preparedness scores across all clinics**.

Mean scores for the research preparedness components were Patient Engagement (3.6, SD 0.8), Data Management and Integration (2.0, SD 1.1), Collaboration and Networking (1.8, SD 1.6), Research Capabilities (1.8, SD 1.0), and Finance and Administrative Support (1.1, SD 1.2) as shown in Fig. 4.

### Data management and integration

Thirty-three percent (n = 81) of clinics reported adequate staffing (>75% of required staff) for research data recording. Electronic integration with external centres was reported as full or majority integration by 27% (n = 67) of clinics, while 31% (n = 76) reported no integration. Regarding data security, 28% (n = 68) of clinics reported no ability to store study data securely.

### Patient engagement

Seventy-six percent (n = 186) of clinics reported scheduling four or more routine visits per year, and 19% (n = 47) reported two to three visits. Conversely, only 5% (n = 11) reported annual or no regular follow-up.

### Research capabilities

GCP certification was held by at least half of the staff in 39% (n = 96) of clinics, while 46% (n = 112) reported certification in fewer than a quarter of staff. Written guidelines for digital data handling were present and followed in 28% (n = 69) of clinics, while 40% (n = 97) had no such guidelines. Regarding ethics oversight, 36% (n = 89) used a local IRB or ethics committee, 22% (n = 53) an affiliate IRB, and 42% (n = 102) reported no IRB access. Quality assurance procedures were present and followed in 41% (n = 101) of clinics.

### Finance and administrative support

Twenty-nine percent (n = 71) of clinics reported previously receiving external research funding. For administrative support, 50% (n = 122) of clinics reported that staff could allocate fewer than 4 h per week to research tasks, while 11% (n = 26) reported more than 16 h.

### Collaboration and networking

Thirty-nine percent (n = 95) of clinics did not participate in any diabetes or research networks, 34% (n = 84) participated in national networks only, and 27% (n = 65) participated in both national and international networks.

Detailed question results by country are provided in Supplementary Tables S3–S5.

## Discussion

This assessment of 244 paediatric and adolescent T1D clinics across 21 LMICs reveals a landscape of moderate yet highly variable readiness. Mean scores of 2.5 for Service Provision and 1.8 for Research Preparedness indicate that while most clinics possess core components necessary for the diagnosis and basic management of T1D (such as clinical examinations, HbA1c testing, and patient education), comprehensive capacity remains elusive. Crucially, the substantial variation between and within countries demonstrates that readiness cannot be inferred from national context alone. This heterogeneity, while offering a diverse setting for the planned Global T1D Cohort Study, necessitates a tailored, clinic-specific approach to capacity building rather than a uniform implementation model.

In the Service Provision domain, clinics demonstrated strong foundations but major gaps in advanced care and staffing. Accessibility and resource management scored highly, and routine interventions like HbA1c testing and complication screening were widely available. However, scores may overstate effective capacity; specialised diagnostics (e.g., autoantibodies, C-peptide, genetic testing) and technologies (e.g., CGM) were unevenly available and often financially inaccessible. Furthermore, multidisciplinary care remains a key target for capacity building.

Workforce limitations were pronounced; only 35% of included clinics reported access to a paediatric endocrinologist/diabetologist—a key gap given the distinct developmental, metabolic, and psychosocial needs of the paediatric T1D population. This likely reflects limited training pathways in many of the included countries, with few offering recognised fellowships in the speciality, often requiring physicians to seek fellowship training abroad if they want to pursue formal paediatric endocrinology training. Although no reliable and systematic data exist on the number of paediatric endocrinologists in LMICs, stakeholder reports from some study countries suggest very small national workforces (e.g., 10–30 paediatric endocrinologists in countries of 30–60 million population), and a 2023 conference abstract estimated the presence of 19 paediatric endocrinologists total across 21 francophone countries in sub-Saharan Africa.^9^ While 75% of clinics employed a nurse, fewer than half (45%) retained a specialist nurse trained in diabetes or endocrinology. Although reported provider-to-patient ratios appeared favourable (69% < 25:1), this metric likely masks the true burden on staff, as nearly 40% of clinics could not provide the staffing counts required to calculate it. This item non-response—likely stemming from optional survey fields being skipped due to a lack of readily available records—underscores a critical need to strengthen digital patient registries alongside workforce upskilling. Additionally, this metric does not account for the burden of other conditions the providers are expected to see, as many centres and clinics will treat endocrine or paediatric conditions broadly.

In the Research Preparedness domain, the primary barriers were structural rather than related to patient contact. Scheduled visit frequency was a relative strength, with most clinics reporting four or more visits per year, which may support retention rates for longitudinal studies, although it is important to note that this may not accurately reflect appointment attendance by patients. Further, this potential is bottlenecked by deficits in governance and infrastructure: many clinics lacked local ethics committee availability, secure interoperable data systems, and protected time for research staff. The lower relative scores in these areas reflect gaps that likely have the greatest practical impact on research participation. These findings suggest that clinics in this study are well-positioned to recruit participants but require substantial investment in digital infrastructure and research administration to participate equitably in global studies.

This study has several limitations. First, as a cross-sectional survey relying on self-reported data from senior clinicians, findings may be subject to recall or social desirability bias, though structured scoring bands were used to mitigate free-text subjectivity, and participants were informed their individual responses would not be made available outside of those conducting the data analysis. Second, recruitment via CDiC networks may favour more engaged or better resourced clinics, potentially overestimating general readiness and limiting generalisability due to the overrepresentation of CDiC centres in the sample. Third, READY-T1D measures infrastructure availability rather than utilisation or clinical outcomes and has not yet undergone formal validation. While the tool was designed through an evidence-based process and grounded in the validated WHO SARA framework and refined following a substantial pilot study (Supplementary File S1), formal validation is a planned step in future research by the group to link readiness scores to endpoints such as HbA1c or DKA rates. Fourth, some conceptual overlap may exist between domains in the tool. Fifth, READY-T1D was designed to capture broad clinic-level capacity and does not address all paediatric-specific dimensions of diabetes care and may exclude some important factors of T1D care (such as transition planning from paediatric to adult care or glucagon access). Finally, the equal-weighting scoring framework provides a broad overview but may not reflect the relative clinical importance of specific components in different contexts, as such, interpretation should include consultation of Table 1 and Supplementary Tables S3–S5, which provide response counts by question overall and by country.

Notwithstanding these limitations, to our knowledge the study represents one of the largest multi-country assessments of clinic-level readiness for paediatric T1D care and research conducted to date, and the results have direct implications for the Global T1D Cohort Study and for policy makers, funders, and stakeholders involved in the design and implementation of T1D services for children and adolescents in LMICs.

READY-T1D findings have already directly informed the design of educational and translations activities of GC-CDiC, with the inclusive design and development of an online training course involving 12 collaborating clinical co-investigators from Asia and Africa and 12 Harvard team members. It spans 20 modules in subject areas for research and clinical training and is designed for researchers and clinicians working in GC-CDiC collaborating clinics, ensuring that training targets these specific high-value gaps.^10^ To date over 100 researchers and clinicians have completed the training modules. Online training has been combined with four in-person workshops in Africa, Asia, Europe, and the Americas involving over 300 researchers and clinicians. Future analyses of READY-T1D data will also include stratified analyses, by facility type and CDiC affiliation, which will strengthen the cross-country comparability of results.

The findings suggest that capacity building must be sequenced to leverage strong patient engagement while prioritising digital transformation, ethics governance, and GCP training. The marked within-country heterogeneity supports a hub-and-spoke model, where higher-scoring clinics act as mentors while lower-scoring sites receive phased support in basic laboratory and inventory management.

READY-T1D provides standardised, comparable data on paediatric and adolescent T1D clinic readiness across 21 GC-CDiC countries. The data identify a clear opportunity: clinics possess core elements for patient access but are constrained by specific deficits in multidisciplinary staffing, digital infrastructure, and research governance. By quantifying these gaps, READY-T1D offers a practical framework to guide targeted capacity-building investments in support of the Global T1D Cohort Study and improved care in the studied settings.

## Contributors

Conception and design: RA, JTF, and CLR. Methodology: RA, JTF, CLR, ZS, HP, MY, and AMD. Data collection: JTF, HP, SA, BA, AA, PA, AB, KL, AMD, VCD, VHN, NTH, ZI, AK, PKKM, IM, BM, CM, JCM, MNM, BN, NMN, MP, AP, VSRD, MPNS, AFS, MS, OSS, SNSS, GT, NT, SAD, LMCS, YC, NY, AU, PNW, MYJ, BZ, BEF, MC, and ZS. Data analysis: HP and JTF. Data interpretation: JTF, HP, CLR, and RA. Writing: JTF, HP, and CLR. JTF and HP accessed and verified the underlying data. Critical review of the manuscript: All authors. Final approval of the manuscript: All authors. Accountable for all aspects of the work: All authors.

## Data sharing statement

Data collected for this study can be made available to researchers in a deidentified format on reasonable request and after signing appropriate data sharing agreements. Requests for data access should be directed to the corresponding author and must be approved by the relevant ethics committees and data custodians.

## Declaration of interests

GC-CDiC is funded by an unrestricted grant from Novo Nordisk. JTF, HP, BF, MJC, ZJW, CLR, and RA declare funding from Novo Nordisk as a grant to institution. MYJ declares funding from Novo Nordisk for CDiC, of which he is the CDiC Malaysia co-chair, funding from Dexcom as a key opinion leader, and funding from Medtronic for an insulin pump workshop.
