# Supplementary material for: READY-T1D–assessment of Research and Service Delivery Readiness for paediatric Type 1 Diabetes: a multi-country cross-sectional study
Source: eClinicalMedicine. 2026 Jul 9;97:104067. doi: 10.1016/j.eclinm.2026.104067 (PMC13380773; doi:10.1016/j.eclinm.2026.104067)
Supplement: Supplementary File S1 [file mmc1.pdf]

Pilot Study Survey and Results

Pilot READY-T1D Survey:

**Service provision:**<br>Clinical Expertise and Staffing	
	Please indicate which of the below resources are available at your centre for children with T1D?<br>(select all that apply)<br><br>* Endocrinologist/Diabetologist<br>* Physician<br>* Dietician<br>* Specialist Nurses<br>* Social Worker<br>* Counsellor<br>* Psychologist
	Do the staff at the clinic have experience in managing Type 1 diabetes cases?
Health Services	
	What proportion of children and adolescents with T1D at your clinic receive comprehensive healthcare services in relation to T1D (diagnosis, treatment and routine management?
	What proportion of children and adolescents receiving these health services, receive these services without paying at the point of care?
	Please indicate which of the below services to screen for T1D complications are provided at your centre for children with T1D?<br>(select all that apply)<br><br>* Clinical exam<br>* Eye fundus<br>* Foot exam<br>* Renal function<br>* Lipid profile<br>* ECG
	Is Diabetic Ketoacidosis managed at your facility?
	What form of management for T1D do most (>50%) children and adolescents with T1D use?<br>(select all that apply)<br>* Subcutaneous Insulin Syringe<br>* Subcutaneous Insulin pen<br>* Insulin Pump

	What equipment and supplies do you provide at your facility to enable children and adolescents to check their blood glucose levels?
	Are there private spaces available for patient consultations?
	Does your facility provide any health prevention or promotion health services for T1D?
	Is there availability of telehealth capability for patient management?
Resource Management	
	What digital devices are currently in place to manage patient data?
	Are the software programs available and up-to-date for collecting and storing digital patient information?
	Does your facility have adequate inventory of Insulin and glucose measuring equipment and supplies to provide to children and adolescents with T1D?
	Does your clinic you have software for inventory management and optimization?
	Is the clinic equipped with stable and secure internet and mobile connectivity for data transmission and storage?
Testing Facilities	
	What routine lab testing are done at the clinic for T1D patients? (HbA1c, Creatinine, TFT, Blood Count)
	Which of these additional testing is available or accessed by the clinic?<br>(select all that apply)<br><br>* Oral glucose tolerance testing<br>* Pancreatic autoantibody<br>* C-peptide testing
	Does the facility have access to genetic testing and utilize this for T1D patients?
	Does your facility have the necessary equipment and supplies to provide support for patients with DKA?
Accessibility	
	Is the clinic accessible to patients with disabilities?
	Is the clinic equipped to provide language support for diverse patient populations?
**Research Preparedness:**	
Data Management & Integration	

	Is the clinic adequately staffed and available for patient data recording for research activities?
	Have staff members received training on using digital systems for data collection?
	Can the clinic's systems integrate digital patient data across various platforms or systems, ensuring seamless access for healthcare providers?
	Does the clinic have the ability to collect and store study-related data securely?
Patient Engagement	
	Are patients adequately informed about the types of data collected and how it will be used?
	Does the clinic conduct regular follow up with T1D patients
Research Capabilities	
	Do the research staff have GCP (Good Clinical Practice) certification?
	Do the staff have experience in conducting clinical research studies related to T1D?
	Are there established guidelines for securely handling and storing digital patient data?
	Do staff members understand the importance of maintaining patient data privacy and confidentiality in a digital environment?
	Do you have an Institutional Ethics committee/Independent Ethics committee in place?
	Are there quality assurance procedures for data collection, documentation, and reporting?
Financial & Administrative Support	
	Is there funding or grants available to support research activities?
	Is there administrative support available for research management and regulatory submissions?
Collaboration and Networking	
	Does the clinic collaborate with other healthcare institutions and academic centres?
	Does the clinic participate in national or international diabetes research networks?

Service provision:	Score - 0 (Low Provision)	Score - 1 (Moderate Provision)	Score - 2 (Full Provision)
**Clinical Expertise and Staffing**			
Please indicate which of the below resources are available at your centre for children with T1D?<br>(select all that apply)<br><br>* Endocrinologist/Diabetologist<br>* Physician<br>* Dietician<br>* Specialist Nurses<br>* Social Worker<br>* Counsellor<br>* Psychologist	Less than 3 resources available	3 resources available	4 or more resources available
Do the staff at the clinic have experience in managing Type 1 diabetes cases?	Less than 25% of the staff have 3 years or more experience in managing T1D cases	25% - 75% of the staff have 3 years or more experience in managing patients with T1D	More than 75% of the staff have 3 years or more experience in managing patients with T1D
**Health Services**			
What proportion of children and adolescents with T1D at your clinic receive comprehensive healthcare services in relation to T1D (diagnosis, treatment and routine management?	Some children and adolescents (<50%)	Most children and adolescents (50-75%)	Almost all children and adolescents (75-100%)
What proportion of children and adolescents receiving these health services, receive these services without paying at the point of care?	Some children and adolescents (<50%)	Most children and adolescents (50-75%)	Almost all children and adolescents (75-100%)
Please indicate which of the below services to screen for T1D complications are provided at your centre for children with T1D?<br>(select all that apply)	Less than 3 choices selected	3 choices selected	4 or more choices selected

* Clinical exam<br>* Eye fundus<br>* Foot exam<br>* Renal function<br>* Lipid Profile<br>* ECG			
Is Diabetic Ketoacidosis managed at your facility?			
What form of management for T1D do most (>50%) children and adolescents with T1D use?<br>(select all that apply)<br>* Subcutaneous Insulin Syringe<br>* Subcutaneous Insulin pen<br>* Insulin Pump	Subcutaneous Insulin Syringe	Subcutaneous Insulin pen	Insulin Pump
What equipment and supplies do you provide at your facility to enable children and adolescents to check their blood glucose levels?	None	Glucometer + Test Strips	Continuous Glucose Monitoring Device
Are there private spaces available for patient consultations?	No	Shared space	Private area
Does your facility provide any health prevention or promotion health services for T1D?	No health prevention or promotion	Health prevention or health promotion	Health prevention and health promotion
Is there availability of telehealth capability for patient management?	No		Yes
**Resource Management**			
What digital devices are currently in place to manage patient data?	No computer or mobile phone/tablet is available	Either computers or mobile phone/tablet is available, but not both	Both computers and mobile phone/tablet are available
Are the software programs available and up-to-date for collecting and storing digital patient information?	There are no software programs available for collecting and storing digital patient information	There are software programs available for collecting and storing digital patient information, but patient data is not up-to-date	There are software programs available for collecting and storing digital patient information, and patient data is up-to-date

Does your facility have adequate inventory of Insulin and glucose measuring equipment and supplies to provide to children and adolescents with T1D?	Stock outs are very common (67%-100%)	Stock outs are frequent (34-66%)	Stock outs are rare (<33%)
Does your clinic you have software for inventory management and optimization?	No inventory management is done either manually or with software	Inventory management is done manually (on paper)	Utilize software for inventory management and optimization
Is the clinic equipped with stable and secure internet and mobile connectivity for data transmission and storage?	Clinic does not have internet/Wi-Fi connectivity or mobile (3G/4G/LTE) connectivity for data transmission and storage	Either internet/Wi-Fi connectivity or mobile connectivity is available at clinic for data transmission and storage	Both internet/Wi-Fi connectivity and mobile (3G/4G/LTE) connectivity is available for data transmission and storage
**Testing Facilities**			
What routine lab testing are done at the clinic for T1D patients?<br>(HbA1c, Creatinine, TFT, Full Blood Count)	1 or less choices selected	2 choices selected	3 or more choices selected
Which of these additional testing is available or accessed by the clinic? (select all that apply)<br><br>* Oral glucose tolerance testing<br>* Pancreatic autoantibody<br>* C-peptide testing	1 or less choices selected	2 choices selected	3 or more choices selected
Does the facility have access to genetic testing and utilize this for T1D patients?	No	Can refer to other facility	Yes
Does your facility have the necessary equipment and supplies to provide support for patients with DKA?	No	Some	All
**Accessibility**			
Is the clinic accessible to patients with disabilities?	Less 25% of services offered is	25% -75% of services offered is accessible	More than 75% of services offered is

	accessible by patients with disabilities	by patients with disabilities	accessible by patients with disabilities
Is the clinic equipped to provide language support for diverse patient populations?	Clinic is not equipped with any services for local language support	Clinic is equipped with support for one local language	Clinic is equipped with support for more than one local language
**Research Preparedness:**	Score - 0 (Low Preparedness)	Score - 1 (Moderate Preparedness)	Score - 2 (Full Preparedness)
**Data Management & Integration**			
Is the clinic adequately staffed and available for patient data recording for research activities?	Less than 25% of the required staff is available for completing patient data recording	25% - 50% of the required staff is available for completing patient data recording	75% of the required staff is available for completing patient data recording
Have staff members received training on using digital systems for data collection?	Less than 25% of the staff know to use Dure Tech application for data collecting and storing	25% - 50% of the staff know to use Dure Tech application for data collecting and storing	More than 50% of the staff know to use Dure Tech application for data collecting and storing
Can the clinic's systems integrate digital patient data across various platforms or systems, ensuring seamless access for healthcare providers?	0% of the clinic's systems integrate digital patient data across various platforms or systems, ensuring seamless access for healthcare providers	50% of the clinic's systems integrate digital patient data across various platforms or systems, ensuring seamless access for healthcare providers	More than 50% of the staff know to use Dure Tech application for data collecting and storing
Does the clinic have the ability to collect and store study-related data securely?	There is no ability to collect or store study-related data securely	The clinic is equipped to collect and store study-related data but no data security is available	The clinic is equipped to collect and store study-related data securely
**Patient Engagement**			
Are patients adequately informed about the types of data collected and how it will be used?	Patients are not informed about what data is being collected	Patients are informed of what data is being collected but not how the data is used	Patients are informed of what data is being collected and how it is used

Does the clinic conduct regular follow up with T1D patients	Patient follow-ups are done every year or longer<br>No patient follow-ups are done	Patient follow-ups are done at least every 6 months	Patient follow-ups are done at least every 3 months
**Research Capabilities**			
Do the research staff have GCP (Good Clinical Practice) certification?	No		Yes
Do the staff have experience in conducting clinical research studies related to T1D?	Less than 25% of the required staff are trained to conduct clinical research studies	25% - 75% of the required staff are trained to conduct clinical research studies	More than 75% of the required staff are trained to conduct clinical research studies
Are there established guidelines for securely handling and storing digital patient data?	No guidelines exist for patient data handling and storing of patient data	Guidelines exist for patient data handling and storing but are not followed	Guidelines exist for patient data handling and storing and are followed in the clinic
Do staff members understand the importance of maintaining patient data privacy and confidentiality in a digital environment?	Staff does not understand the importance of data privacy	Staff understands data privacy but do not follow the guidelines	Staff understands data privacy and follow the guidelines
Do you have an Institutional Ethics committee/Independent Ethics committee in place?	No		Yes
Are there quality assurance procedures for data collection, documentation, and reporting?	No procedures exist for quality assurance	Procedures exist for quality assurance but are not followed	Procedures exist for quality assurance and are followed
**Financial & Administrative Support**			
Is there funding or grants available to support research activities?	Less than 25% of the funds needed are available	25% - 75% of the funds needed are available	More than 75% of the funds needed are available
Is there administrative support available for research management and regulatory submissions?	Less than 25% of the administrative staff needed are available	25% - 75% of the administrative staff needed are available	More than 75% of the administrative staff needed are available
**Collaboration and Networking**			
Does the clinic collaborate with other healthcare	The clinic does not collaborate with	The clinic collaborates with	The clinic collaborates with

institutions and academic centres?	healthcare institutions or academic centres?	either healthcare institutions or academic centres, but not both	both healthcare institutions and academic centres
Does the clinic participate in national or international diabetes research networks?	The clinic does not participate in national or international diabetes research networks	The clinic participates in national but not international diabetes research networks	The clinic participates in both national and international diabetes research networks

Pilot READY-T1D Results

All countries

**All Countries**		
**No.**	**Assessment Component**	**Score**
**1**	**Service Provision**	**1.08**
1	Clinical Expertise and Staffing	1.20
1	Health Services	1.09
1	Resource Management	0.94
1	Testing Facilities	0.64
2	Accessibility	1.54
**2**	**Research Preparedness**	**0.94**
2	Data Management & Integration	0.63
2	Patient Engagement	1.48
2	Research Capabilities	0.99
2	Financial and Administrative Support	0.37
2	Collaboration and Networking	1.25

Cameroon

**Cameroon**		
	**Assessment Component**	**Score**
**1**	**Service Provision**	**1.01**
1	Clinical Expertise and Staffing	1.50
1	Health Services	1.07
1	Resource Management	0.80
1	Testing Facilities	0.50
2	Accessibility	1.17
**2**	**Research Preparedness**	**1.14**
2	Data Management & Integration	0.67
2	Patient Engagement	1.67

2	Research Capabilities	1.06
2	Financial and Administrative Support	0.33
2	Collaboration and Networking	2.00

Ethiopia

Ethiopia		
	**Assessment Component**	**Score**
**1**	**Service Provision**	**0.93**
1	Clinical Expertise and Staffing	0.84
1	Health Services	1.00
1	Resource Management	0.58
1	Testing Facilities	0.42
2	Accessibility	1.82
**2**	**Research Preparedness**	**0.75**
2	Data Management & Integration	0.25
2	Patient Engagement	0.94
2	Research Capabilities	0.56
2	Financial and Administrative Support	0.87
2	Collaboration and Networking	1.15

Guinea

Guinea		
	**Assessment Component**	**Score**
**1**	**Service Provision**	**0.96**
1	Clinical Expertise and Staffing	1.14
1	Health Services	1.22
1	Resource Management	0.83
1	Testing Facilities	0.18
2	Accessibility	1.43
**2**	**Research Preparedness**	**0.86**
2	Data Management & Integration	0.79
2	Patient Engagement	1.93
2	Research Capabilities	0.88
2	Financial and Administrative Support	0.00
2	Collaboration and Networking	0.71

India

India		
	**Assessment Component**	**Score**
**1**	**Service Provision**	**1.36**
1	Clinical Expertise and Staffing	1.25
1	Health Services	1.27
1	Resource Management	1.52

1	Testing Facilities	1.06
2	Accessibility	1.70
**2**	**Research Preparedness**	**1.09**
2	Data Management & Integration	1.02
2	Patient Engagement	1.54
2	Research Capabilities	1.27
2	Financial and Administrative Support	0.42
2	Collaboration and Networking	1.20

Malaysia

**Malaysia**		
	**Assessment Component**	**Score**
**1**	**Service Provision**	**1.15**
1	Clinical Expertise and Staffing	1.28
1	Health Services	0.89
1	Resource Management	0.97
1	Testing Facilities	1.03
2	Accessibility	1.57
**2**	**Research Preparedness**	**0.86**
2	Data Management & Integration	0.42
2	Patient Engagement	1.30
2	**Service Provision**	1.18
2	Financial and Administrative Support	0.22
2	Collaboration and Networking	1.17
