# Supplementary material for: READY-T1D–assessment of Research and Service Delivery Readiness for paediatric Type 1 Diabetes: a multi-country cross-sectional study
Source: eClinicalMedicine. 2026 Jul 9;97:104067. doi: 10.1016/j.eclinm.2026.104067 (PMC13380773; doi:10.1016/j.eclinm.2026.104067)
Supplement: Supplementary File S2 [file mmc2.pdf]

Summary of Incomplete Surveys

Number of incomplete surveys at each percentage of completion:

Completion Percentage	n
0%	8
2%	143
27%	3
29%	3
48%	3
65%	1
77%	3
Total	*164*

Surveys with ≤2% completion matched to surveys via IP addresses in metadata:
Surveys with ≤2% completion did not include any information input by participants. These likely represent survey links there were opened to check how long the survey may take to complete and/or to identify any survey questions they may need additional information to appropriately answer. The survey software automatically collects IP addresses from most respondents, which was able to be used to link incomplete surveys with completed surveys, or with other incomplete surveys. Incomplete surveys matched to other incomplete surveys were further grouped to identify how many unique IP addresses they comprised – which could be considered as additional 'not able to match' surveys.

<u>Total: 151</u>
Matched to completed surveys: 132 (87%)
Matched to other incomplete surveys (but not able to match to completed survey): 13 (9%)
- Of which were unique IP addresses: 5

Not able to match: 6 (4%)

Surveys with 27-77% completion matched to surveys:
These surveys frequently contained only basic demographic information (country, clinic name and address, clinician name). Those with 48-77% completion contained responses to some survey questions beyond demographics. These likely represent surveys where the survey software was closed before completion due to network/device issues, accidental closure, or closure with the intention of returning to complete later. These surveys were compared first with surveys from the same listed country to identify any matches based on clinic or clinician name. The remaining unmatched surveys were also compared using IP addresses.

<u>Total: 13</u>
Matched to completed surveys: 12 (92%)
Not able to match: 1 (8%)

The survey that could not be match to a survey completed later had 27% completion.
