# Supplementary material for: READY-T1D–assessment of Research and Service Delivery Readiness for paediatric Type 1 Diabetes: a multi-country cross-sectional study
Source: eClinicalMedicine. 2026 Jul 9;97:104067. doi: 10.1016/j.eclinm.2026.104067 (PMC13380773; doi:10.1016/j.eclinm.2026.104067)
Supplement: Supplementary Tables S1–S5 [file mmc3.pdf]

Supplementary Table S1: READY-T1D Survey Questions and Scoring System

Section	Survey Question	Response Options	Scoring Type	Scoring Method	Score Calculation	Conditional Logic	Notes
Normalization: 0-4 scale per metric where 0 = lowest readiness, 4 = highest readiness							
**DESCRIPTIVE INFORMATION**	Do you Consent to Participate in this Study	* Yes - I consent<br>* No - I do not consent	Not Scored	Descriptive	N/A		
	Name of the clinic	Free text	Not Scored	Descriptive	N/A		
	Address of the clinic (street and town/city)	Free text	Not Scored	Descriptive	N/A		
	Post code/zip code of clinic	Free text	Not Scored	Descriptive	N/A		
	Country of Clinic	Free text	Not Scored	Descriptive	N/A		
	Clinician's name	Free text	Not Scored	Descriptive	N/A		
	Clinician's email	Free text	Not Scored	Descriptive	N/A		
	Clinician's phone number (optional)	Free text	Not Scored	Descriptive	N/A		
	Please indicate which one of the following does your centre belong to	* CDIC centre<br>* CDIC Satellite centre<br>* Non-CDIC centre	Not Scored	Descriptive	N/A		
	Is your clinic/centre public, private, or mixed?	* Public<br>* Private<br>* Mixed	Not Scored	Descriptive	N/A		
	Does your clinic/centre provide inpatient care, outpatient care, or both?	* Inpatient only<br>* Outpatient only<br>* Both inpatient and outpatient	Not Scored	Descriptive	N/A		
	Please indicate what type of clinic/centre yours is	* Primary Care<br>* Urgent Care<br>* Community Health Center<br>* Specialty Outpatient Center<br>* General Hospital<br>* District General Hospital/Regional Hospital<br>* Teaching/Academic Hospital<br>* Tertiary/Quaternary Hospital<br>* None of the above	Not Scored	Descriptive	N/A		

	Approximately how many people of ANY age with T1D do you have registered to your clinic?	Free text (number)	Not Scored	Descriptive	N/A		
	Approximately how many children (≤20 years old) with T1D do you have registered to your clinic?	Free text (number)	Not Scored	Descriptive	N/A		Used for provider-to-patient ratio calculation
**CLINICAL EXPERTISE AND STAFFING**	**Q1**<br><br>Please indicate which of the below practitioners are available at your centre to treat T1D in patients ≤20 years old and how many (FT=1, PT=0.5)	* Endocrinologist/Diabetologist<br>* Paediatric endocrinologist/ diabetologist<br>* Paediatrician (non-endocrinologist)<br>* General Physician/Non-Specialist<br>* Trainee physician<br>* Dietician<br>* Specialist Nurses<br>* Nurses<br>* Medical Assistants/Health Care Assistants<br>* Social Worker<br>* Psychologist/Counsellor	SCORED - Split Metric	Normalized Count (Variety)	(Number of TYPES selected / 11) × 4		Split into 2 metrics. Metric 1A scores variety of expertise types available
	[SAME AS ABOVE - Second metric from same question]	[Same response used for calculation]	SCORED - Split Metric	Provider-to-Patient Ratio	Total FTE providers / Children with T1D. Score based on ratio: <1:100=0pts, 1:100-1:75=1pt, 1:75-1:50=2pts, 1:50-1:25=3pts, >1:25=4pts	Requires patient count from descriptive questions	1B scores adequacy of staffing relative to patient load
**HEALTH SERVICES**	**Q2**<br><br>Please indicate which services to screen for T1D complications are routinely provided (select all)	* Clinical Exam<br>* Eye fundus exam<br>* Foot exam<br>* Height and weight measurement	SCORED	Normalized Count	(Number selected / 4) × 4		
	**Q3**<br><br>What equipment do most (>50%) children use to check blood glucose?	* None<br>* Glucometer + Test Strips + Syringes<br>* Continuous Glucose Monitoring Device	SCORED	Ranked Options	None=0.0, Glucometer=2.0, CGM=4.0		

	**Q4**<br><br>What equipment do you PROVIDE for blood glucose/ketone monitoring (free or paid)?	* None<br>* For Free: Glucometer<br>* For Free: Test strips<br>* For Free: CGM<br>* For Free: Capillary Ketone monitor<br>* For Free: Urine Ketone Strips<br>* Paid: Glucometer<br>* Paid: Test strips<br>* Paid: CGM<br>* Paid: Capillary Ketone monitor<br>* Paid: Urine Ketone Strips	SCORED - Split Metric	Equipment Availability (Any payment type)	(Number of unique equipment types available / 5) × 4. Count each equipment type once regardless of free/paid.		
	**Q5**<br><br>What insulin delivery do most (>50%) children use?	* Subcutaneous Insulin Syringe<br>* Subcutaneous Insulin pen<br>* Insulin Pump	SCORED	Ranked Options	Syringe=0.0, Pen=2.0, Pump=4.0		
	**Q6**<br><br>Is acute DKA managed at your facility? (from diagnosis to discharge)	* No<br>* Only diagnosis<br>* Yes - From diagnosis to treatment to discharge	NOT SCORED - Descriptive/Conditional Trigger	Descriptive	N/A	Determines if next question applies	Descriptive. Used to determine facility capability tier. Outpatient clinics should not be penalized.
	**Q7**<br><br>Which of the following does your clinic have to treat acute DKA? (select all)	* None<br>* Capillary glucose monitor<br>* Capillary ketone monitor<br>* POC blood gas analysis<br>* Continuous cardiac monitoring/pulse oximetry<br>* Fast-acting insulin<br>* IV fluids<br>* Potassium replacement	CONDITIONAL SCORED	Binary threshold (Conditional)	Score = 4.0 if all four required DKA treatment capacities are present; 0.0 otherwise.	Only scored if Q6 = "Yes - From diagnosis to treatment to discharge". Required capacities (from Q7) are: (1) monitoring (Capillary glucose monitor or POC blood gas analysis), (2) fast-acting or short-acting insulin, (3) IV fluids, and (4) potassium replacement.	Binary DKA treatment readiness metric. Clinics that do not fully manage DKA (Q6 not "Yes - From diagnosis to treatment to discharge") are coded as N/A and excluded from sub-component means.
	**Q8**<br><br>What proportion receive structured diabetes education at diagnosis?	* 0-25%<br>* 25-50%<br>* 50-75%<br>* 75-100%	SCORED	Ranked Options	0-25%=0.0, 25-50%=1.3, 50-75%=2.7, 75-100%=4.0		
	**Q9**<br><br>What proportion receive services without paying at point of care?	* None<br>* <25%<br>* 25-50%<br>* 50-75%<br>* 75-<100%<br>* 100%	SCORED	Ranked Options	None=0.0, <25%=0.8, 25-50%=1.6, 50-75%=2.4, 75-<100%=3.2, 100%=4.0		
	**Q10**<br><br>Are there private spaces for patient consultations?	* No<br>* Yes	SCORED	Binary	No=0.0, Yes=4.0		

	**Q11**<br><br>Is telehealth capability available?	* No<br>* Yes	SCORED	Binary	No=0.0, Yes=4.0		
**RESOURCE MANAGEMENT**	**Q12**<br><br>What digital devices are in place to manage patient data?	* No computer or mobile device<br>* Either computers OR mobile devices<br>* Both computers AND mobile devices	SCORED	Ranked Options	None=0.0, Either=2.0, Both=4.0		
	**Q13**<br><br>Are software programs available and up to date for digital patient information?	* No software available<br>* Software available but not up to date<br>* Software available and up to date	SCORED	Ranked Options	No software=0.0, Not up to date=2.0, Up to date=4.0		
	**Q14**<br><br>How often does your facility experience insulin stock outs?	* Insulin not stocked/provided<br>* Stock outs >30% of year (>120 days)<br>* Stock outs 20-30% (81-120 days)<br>* Stock outs 15-20% (61-80 days)<br>* Stock outs 10-15% (41-60 days)<br>* Stock outs 5-10% (20-40 days)<br>* Stock outs 0-5% (0-19 days)	CONDITIONAL SCORED	Reverse-Ranked (Conditional)	If "Not stocked"=N/A (exclude). Otherwise: >30%=0.0, 20-30%=0.8, 15-20%=1.6, 10-15%=2.4, 5-10%=3.2, 0-5%=4.0	If "Not stocked/provided" selected, mark N/A and EXCLUDE from averaging	"Not stocked" triggers N/A instead of 0 score. Appropriate for clinics where patients source externally.
	**Q15**<br><br>How often does your facility experience glucose equipment stock outs?	* Equipment not stocked/provided<br>* Stock outs >30% of year (>120 days)<br>* Stock outs 20-30% (81-120 days)<br>* Stock outs 15-20% (61-80 days)<br>* Stock outs 10-15% (41-60 days)<br>* Stock outs 5-10% (20-40 days)<br>* Stock outs 0-5% (0-19 days)	CONDITIONAL SCORED	Reverse-Ranked (Conditional)	If "Not stocked"=N/A (exclude). Otherwise: >30%=0.0, 20-30%=0.8, 15-20%=1.6, 10-15%=2.4, 5-10%=3.2, 0-5%=4.0	If "Not stocked/provided" selected, mark N/A and EXCLUDE from averaging	"Not stocked" triggers N/A instead of 0 score. Appropriate for clinics where patients source externally.
	**Q16**<br><br>Does your facility have software for inventory management?	* No inventory management<br>* Manual inventory management<br>* Software-based inventory management	SCORED	Ranked Options	None=0.0, Manual=2.0, Software=4.0		
	**Q17**<br><br>Is the clinic equipped with stable internet connectivity?	* No internet<br>* Intermittent internet<br>* Internet 50-75% of time<br>* Internet >75% of time	SCORED	Ranked Options	No internet=0.0, Intermittent=1.3, 50-75%=2.7, >75%=4.0		
**TESTING FACILITIES**	**Q18**<br><br>Which lab tests are available for T1D patients? (select all)	* None<br>* HbA1c<br>* Lipids<br>* Serum Creatinine<br>* Urine Creatinine	SCORED	Normalized Count	(Number selected / 6) × 4		

		* TFT<br>* Full Blood Count					
	**Q19**<br><br>Which tests are available/accessed? (select all)	* None<br>* Oral glucose tolerance testing<br>* Pancreatic autoantibody<br>* C-peptide testing	SCORED	Normalized Count	(Number selected / 3) × 4		
	**Q20**<br><br>Does the facility have access to genetic testing for T1D patients?	* No<br>* Yes	SCORED	Binary	No=0.0, Yes=4.0		
**ACCESSIBILITY**	**Q21**<br><br>What proportion of T1D patients travel over 60 minutes to reach your centre?	* 66-100%<br>* 33-66%<br>* <33%<br>* None	NOT SCORED - Contextual	Descriptive	N/A		Contextual. This is a health system indicator, not clinic quality. Clinics have no control over geographic access.
	**Q22**<br><br>Is the clinic equipped for language support for diverse populations?	* Not equipped<br>* Support for one local language<br>* Support for multiple languages	SCORED	Ranked Options	Not equipped=0.0, One language=2.0, Multiple=4.0		
**DATA MANAGEMENT AND INTEGRATION**	**Q23**<br><br>Is the clinic adequately staffed for patient data recording for research?	* <25% of required staff<br>* 25-75% of required staff<br>* >75% of required staff	SCORED	Ranked Options	<25%=0.0, 25-75%=2.0, >75%=4.0		
	**Q24**<br><br>Can systems integrate electronic patient records from external health centres?	* No digital systems<br>* No integration (0% of providers)<br>* Some integration (<25%)<br>* Majority integration (25-75%)<br>* Full integration (75-100%)	SCORED	Ranked Options	No systems=0.0, No integration=1.0, Some=2.0, Majority=3.0, Full=4.0		
	**Q25**<br><br>Does the clinic have ability to store study data securely?	* No ability<br>* Ability but done 0%<br>* Done <50%<br>* Done 50-75%<br>* Done >75%	SCORED	Ranked Options	No ability=0.0, 0%=1.0, <50%=2.0, 50-75%=3.0, >75%=4.0		

**PATIENT ENGAGEMENT**	**Q26**<br><br>Does the clinic conduct regular follow-up with T1D patients?	* No regular follow-up<br>* Once a year<br>* 2 times/year (every 6 months)<br>* 3 times/year (every 4 months)<br>* 4+ times/year (every 3 months or more)	SCORED	Ranked Options	No follow-up=0.0, 1x/year=1.0, 2x/year=2.0, 3x/year=3.0, 4+x/year=4.0		
**RESEARCH CAPABILITIES**	**Q27**<br><br>What percentage of research staff have GCP certification?	* <25%<br>* 25-50%<br>* 50-75%<br>* >75%	SCORED	Ranked Options	<25%=0.0, 25-50%=1.3, 50-75%=2.7, >75%=4.0		
	**Q28**<br><br>Do staff have experience in clinical research studies?	* <25%<br>* 25-50%<br>* 50-75%<br>* >75%	SCORED	Ranked Options	<25%=0.0, 25-50%=1.3, 50-75%=2.7, >75%=4.0		
	**Q29**<br><br>Are there established guidelines for handling digital patient data?	* No guidelines<br>* Guidelines exist, followed <50%<br>* Guidelines exist, followed 50-75%<br>* Guidelines exist, followed >75%	SCORED	Ranked Options	No guidelines=0.0, <50%=1.3, 50-75%=2.7, >75%=4.0		
	**Q30**<br><br>Are staff trained in data privacy in digital environment?	* Not trained<br>* <25%<br>* 25-50%<br>* 50-75%<br>* >75%	SCORED	Ranked Options	Not trained=0.0, <25%=1.0, 25-50%=2.0, 50-75%=3.0, >75%=4.0		
	**Q31**<br><br>Do you have an IRB/Ethics Committee locally?	* No IRB<br>* IRB at affiliate centre<br>* Local IRB	SCORED	Ranked Options	No IRB=0.0, Affiliate=2.0, Local=4.0		
	**Q32**<br><br>How frequently does your IRB/Ethics Committee meet?	* No IRB<br>* <once/3 months<br>* Every 2-3 months<br>* Monthly<br>* Weekly	SCORED	Ranked Options	No IRB=0.0, <once/3mo=1.0, Every 2-3mo=2.0, Monthly=3.0, Weekly=4.0		
	**Q33**<br><br>Are there quality assurance procedures for data collection?	* No procedures<br>* Procedures exist but not followed<br>* Procedures exist and followed	SCORED	Ranked Options	No procedures=0.0, Not followed=2.0, Followed=4.0		
**FINANCE AND ADMINISTRATIVE SUPPORT**	**Q34**	* No<br>* Yes	SCORED	Binary	No=0.0, Yes=4.0		

	Have you previously acquired grants/funding for research?						
	**Q35**<br><br>How much time can admin staff allocate to research support (avg hours per staff)?	* <4 hours/week per staff<br>* 4-8 hours/week per staff<br>* 8-12 hours/week per staff<br>* 12-16 hours/week per staff<br>* >16 hours/week per staff	SCORED	Ranked Options	<4 hours/week = 0.0,<br>4-8 hours/week = 1.0<br>8-12 hours/week = 2.0<br>12-16 hours/week = 3.0<br>>16 hours/week = 4.0		
**COLLABORATION AND NETWORKING**	**Q36**<br><br>Does the clinic participate in diabetes research networks?	* Does not participate<br>* National only<br>* Both national and international	SCORED	Ranked Options	Does not participate=0.0,<br>National only=2.0,<br>Both=4.0		

Supplementary Table S2: Survey Response Rate by Country and Overall

**Country**	**Completed Surveys**	**Invited**	**Response rate**
India	51	69	74%
Malaysia	33	40	83%
Ethiopia	32	34	94%
Kenya	28	29	97%
Pakistan	24	24	100%
Bangladesh	7	8	88%
Brazil	7	12	58%
Cambodia	12	12	100%
Cameroon	10	11	91%
Colombia	5	5	100%
Cote d'Ivoire	5	6	83%
Ecuador	1	1	100%
Ghana	1	1	100%
Guinea	8	8	100%
Indonesia	12	16	75%
Morocco	1	1	100%
Niger	1	1	100%
Nigeria	1	1	100%
Peru	3	6	50%
Senegal	1	1	100%
Vietnam	1	1	100%
**All:**	**244**	**287**	**85%**

Supplementary Table S3: Question Results by Country (BGD, BRA, KHM, CMR, COL, CIV, ECU)

Question	Component	BGD		BRA		KHM		CMR		COL		CIV		ECU	
**Clinical Staffing and Experience**		(n = 7)	%	(n = 7)	%	(n = 12)	%	(n = 10)	%	(n = 5)	%	(n = 5)	%	(n = 1)	%
**Q1: Please indicate which of the below practitioners are available at your centre to treat T1D in patients ≤20 years old and how many**	**Nurses**	4	57	3	43	10	83	8	80	2	40	5	100	0	0
	**Endocrinologists/Diabetologists**	7	100	7	100	6	50	6	60	2	40	2	40	1	100
	**Dietician**	7	100	7	100	2	17	8	80	4	80	2	40	1	100
	**General physician/non-specialist**	7	100	1	14	8	67	9	90	2	40	4	80	1	100
	**Paediatrician (non-endo)**	5	71	1	14	4	33	7	70	3	60	3	60	0	0
	**Specialist nurses**	0	0	5	71	2	17	6	60	2	40	2	40	0	0
	**Psychologist/counsellor**	7	100	6	86	2	17	7	70	3	60	1	20	1	100
	**Medical assistants**	7	100	4	57	1	8	4	40	2	40	1	20	1	100
	**Paediatric endocrinologist**	1	14	5	71	2	17	6	60	5	100	2	40	1	100
	**Social worker**	7	100	5	71	1	8	6	60	3	60	2	40	0	0
	**Trainee physician**	0	0	5	71	3	25	5	50	2	40	1	20	0	0
**Health Services**															
**Q2: Please indicate which services to screen for T1D complications are routinely provided**	**Clinical Exam**	7	100	7	100	12	100	9	90	5	100	5	100	1	100
	**Eye Fundus Exam**	1	14	7	100	5	42	2	20	2	40	3	60	1	100
	**Foot Exam**	2	29	7	100	8	67	7	70	2	40	4	80	1	100
	**Height and weight measurement**	6	86	7	100	11	92	10	100	5	100	5	100	1	100
**Q3: What equipment do most (>50%) children use to check blood glucose?**	**Glucometer + test strips**	7	100	6	86	8	67	10	100	2	40	5	100	1	100

	**Continuous Glucose Monitoring Device**	0	0	1	14	4	33	0	0	3	60	0	0	0	0
	**None**	0	0	0	0	0	0	0	0	0	0	0	0	0	0
**Q4: What equipment do you provide for blood glucose/ketone monitoring (free or paid)?**	**For Free: Glucometer**	7	100	7	100	10	83	9	90	3	60	5	100	1	100
	**For Free: Test strips**	7	100	6	86	11	92	10	100	3	60	5	100	1	100
	**For Free: Urine ketone strips**	5	71	1	14	5	42	2	20	1	20	2	40	0	0
	**For Free: Capillary ketone monitor**	4	57	0	0	3	25	2	20	1	20	1	20	0	0
	**For Free: CGM**	4	57	0	0	5	42	2	20	2	40	1	20	0	0
	**Paid: Glucometer**	0	0	1	14	2	17	1	10	2	40	1	20	0	0
	**Paid: Test strips**	0	0	1	14	0	0	0	0	2	40	0	0	0	0
	**Paid: CGM**	2	29	3	43	0	0	1	10	2	40	1	20	1	100
	**Paid: Capillary Ketone Monitor**	0	0	1	14	0	0	0	0	0	0	0	0	0	0
	**Paid: Urine Ketone Strips**	1	14	1	14	2	17	5	50	0	0	2	40	0	0
	**None**	0	0	0	0	0	0	0	0	1	20	0	0	0	0
**Q5: What insulin delivery do most (>50%) children use?**	**Subcutaneous Insulin Syringe**	7	100	0	0	3	25	9	90	0	0	5	100	0	0
	**Subcutaneous Insulin Pen**	0	0	7	100	9	75	1	10	5	100	0	0	1	100
	**Insulin Pump**	0	0	0	0	0	0	0	0	0	0	0	0	0	0
**Q6: Is acute DKA managed at your facility? (from diagnosis to discharge)**	**No**	1	14	0	0	2	17	1	10	3	60	0	0	1	100
	**Only diagnosis**	4	57	1	14	5	42	1	10	0	0	0	0	0	0
	**Yes - From diagnosis to treatment to discharge**	2	29	6	86	5	42	8	80	2	40	5	100	0	0

**Q7: Which of the following does your clinic have to treat acute DKA? (% of those not answering no to Q6)**	**None**	1	17	0	0	1	10	0	0	2	100	0	0	0	0
	**Capillary glucose monitor**	6	100	7	100	10	100	9	100	3	150	4	80	1	0
	**Capillary ketone monitor**	6	100	4	57	4	40	4	44	0	0	1	20	0	0
	**POC blood gas analysis**	3	50	6	86	6	60	1	11	2	100	1	20	0	0
	**Continuous cardiac monitoring/pulse oximetry**	1	17	7	100	8	80	4	44	2	100	1	20	0	0
	**Fast-acting insulin**	1	17	7	100	10	100	8	89	3	150	5	100	1	0
	**IV fluids**	4	67	7	100	9	90	9	100	2	100	5	100	0	0
	**Potassium replacement**	1	17	7	100	5	50	9	100	2	100	4	80	0	0
**Q8: What proportion receive structured diabetes education at diagnosis?**	**0-25%**	0	0	1	14	0	0	2	20	0	0	0	0	0	0
	**25-50%**	0	0	1	14	3	25	1	10	1	20	0	0	0	0
	**50-75%**	4	57	1	14	4	33	1	10	0	0	1	20	0	0
	**75-100%**	3	43	4	57	5	42	6	60	4	80	4	80	1	100
**Q9: What proportion receive services without paying at point of care?**	**None**	0	0	0	0	0	0	0	0	1	20	1	20	0	0
	**<25%**	0	0	0	0	1	8	1	10	2	40	0	0	0	0
	**25-50%**	0	0	0	0	2	17	2	20	0	0	0	0	1	100
	**50-75%**	4	57	0	0	3	25	0	0	0	0	0	0	0	0
	**75-<100%**	0	0	0	0	1	8	2	20	1	20	0	0	0	0
	**100%**	3	43	7	100	5	42	5	50	1	20	4	80	0	0
**Q10: Are there private spaces for patient consultations?**	**Yes**	7	100	4	57	10	83	6	60	5	100	3	60	1	100

	**No**	0	0	3	43	2	17	4	40	0	0	2	40	0	0
**Q11: Is telehealth capability available?**	**Yes**	3	43	6	86	7	58	1	10	5	100	1	20	1	100
	**No**	4	57	1	14	5	42	9	90	0	0	4	80	0	0
**Resource Management**															
**Q12: What digital devices are in place to manage patient data?**	**Both computers and mobile devices**	0	0	2	29	6	50	0	0	4	80	1	20	1	100
	**Either computers or mobile devices**	6	86	5	71	5	42	7	70	1	20	4	80	0	0
	**No computer or mobile device**	1	14	0	0	1	8	3	30	0	0	0	0	0	0
**Q13: Are software programs available and up to date for digital patient information?**	**Software available and up to date**	0	0	5	71	6	50	0	0	3	60	1	20	0	0
	**Software available but not up to date**	5	71	1	14	1	8	4	40	1	20	2	40	1	100
	**No software available**	2	29	1	14	5	42	6	60	1	20	2	40	0	0
			0		0		0		0		0		0		0
**Q14: How often does your facility experience insulin stock outs?**	**Stock outs 0-5% (0-19 days)**	7	100	3	43	7	58	6	60	2	40	3	60	0	0
	**Stock outs 5-10% (20-40 days)**	0	0	0	0	2	17	2	20	2	40	0	0	1	100
	**Stock outs 10-15% (41-60 days)**	0	0	2	29	0	0	1	10	0	0	0	0	0	0
	**Stock outs 15-20% (61-80 days)**	0	0	0	0	1	8	0	0	0	0	1	20	0	0
	**Stock outs 20-30% (81-120 days)**	0	0	1	14	1	8	0	0	0	0	0	0	0	0
	**Stock outs >30% of year (>120 days)**	0	0	0	0	1	8	1	10	0	0	1	20	0	0
	**Insulin not stocked/provided**	0	0	1	14	0	0	0	0	1	20	0	0	0	0

**Q15: How often does your facility experience glucose equipment stock outs?**	**Stock outs 0-5% (0-19 days)**	7	100	4	57	7	58	2	20	4	80	3	60	1	100
	**Stock outs 5-10% (20-40 days)**	0	0	1	14	1	8	2	20	1	20	0	0	0	0
	**Stock outs 10-15% (41-60 days)**	0	0	0	0	0	0	1	10	0	0	0	0	0	0
	**Stock outs 15-20% (61-80 days)**	0	0	0	0	2	17	0	0	0	0	0	0	0	0
	**Stock outs 20-30% (81-120 days)**	0	0	0	0	0	0	3	30	0	0	1	20	0	0
	**Stock outs >30% of year (>120 days)**	0	0	0	0	1	8	1	10	0	0	1	20	0	0
	**Equipment not stocked/provided**	0	0	2	29	1	8	1	10	0	0	0	0	0	0
**Q16: Does your facility have software for inventory management?**	**Software-based inventory management**	0	0	3	43	6	50	0	0	3	60	1	20	1	100
	**Manual inventory management**	6	86	3	43	4	33	6	60	1	20	4	80	0	0
	**No inventory management**	1	14	1	14	2	17	4	40	1	20	0	0	0	0
**Q17: Is the clinic equipped with stable internet connectivity?**	**Internet >75% of time**	3	43	5	71	9	75	1	10	5	100	1	20	1	100
	**Internet 50-75% of time**	3	43	2	29	2	17	1	10	0	0	0	0	0	0
	**Intermittent internet**	1	14	0	0	1	8	2	20	0	0	2	40	0	0
	**No internet**	0	0	0	0	0	0	6	60	0	0	2	40	0	0
**Testing Facilities**															
**Q18: Which lab tests are available for T1D patients?**	**HbA1c**	7	100	7	100	12	100	9	90	5	100	5	100	1	100
	**Lipid profile**	7	100	7	100	10	83	3	30	5	100	3	60	0	0
	**Serum creatinine**	7	100	7	100	11	92	4	40	5	100	5	100	0	0
	**Urine creatinine**	7	100	7	100	7	58	2	20	5	100	2	40	0	0
	**Thyroid function test**	1	14	7	100	6	50	0	0	5	100	0	0	0	0

	**Full blood count**	7	100	7	100	11	92	3	30	5	100	5	100	0	0
	**None**	0	0	0	0	0	0	0	0	0	0	0	0	0	0
**Q19: Which tests are available/accessed?**	**Oral glucose tolerance testing**	7	100	6	86	6	50	3	30	5	100	1	20	0	0
	**Pancreatic autoantibody**	0	0	6	86	3	25	1	10	3	60	1	20	0	0
	**C-peptide testing**	3	43	6	86	5	42	3	30	5	100	1	20	0	0
**Q20: Does the facility have access to genetic testing for T1D patients?**	**Yes**	0	0	1	14	1	8	2	20	1	20	0	0	0	0
	**No**	7	100	6	86	11	92	8	80	4	80	5	100	1	100
**Accessibility**															
**Q21: What proportion of T1D patients travel over 60 minutes to reach your centre?**	**66-100%**	3	43	1	14	1	8	6	60	1	20	3	60	0	0
	**33-66%**	4	57	5	71	7	58	4	40	3	60	2	40	0	0
	**<33%**	0	0	1	14	2	17	0	0	1	20	0	0	1	100
	**None**	0	0	0	0	2	17	0	0	0	0	0	0	0	0
**Q22: Is the clinic equipped for language support for diverse populations?**	**Support for multiple languages**	3	43	1	14	6	50	1	10	2	40	2	40	1	100
	**Support for one local language**	3	43	1	14	0	0	1	10	2	40	0	0	0	0
	**Not equipped**	1	14	5	71	6	50	8	80	1	20	3	60	0	0
**Data Management**															
**Q23: Is the clinic adequately staffed for patient data recording for research?**	**>75% of required staff**	0	0	4	57	2	17	0	0	2	40	1	20	1	100

	**25-75% of required staff**	5	71	2	29	5	42	2	20	2	40	0	0	0	0
	**<25% of required staff**	2	29	1	14	5	42	8	80	1	20	4	80	0	0
**Q24: Can systems integrate electronic patient records from external health centers?**	**Full integration (75-100%)**	0	0	1	14	2	17	0	0	1	20	0	0	0	0
	**Majority integration (25-75%)**	1	14	3	43	3	25	1	10	1	20	0	0	0	0
	**Some integration (<25%)**	4	57	2	29	1	8	0	0	0	0	0	0	0	0
	**No integration (0%)**	1	14	1	14	4	33	4	40	3	60	1	20	0	0
	**No digital systems**	1	14	0	0	2	17	5	50	0	0	4	80	1	100
**Q25: Does the clinic have ability to store study data securely?**	**>75% securely stored**	1	14	4	57	3	25	3	30	5	100	4	80	1	100
	**50-75% securely stored**	2	29	0	0	2	17	0	0	0	0	0	0	0	0
	**<50% securely stored**	3	43	1	14	2	17	2	20	0	0	0	0	0	0
	**Ability but done 0%**	1	14	1	14	0	0	0	0	0	0	0	0	0	0
	**No ability**	0	0	1	14	5	42	5	50	0	0	1	20	0	0
**Patient Engagement**															
**Q26: Does the clinic conduct regular follow-up with T1D patients?**	**≥4 times/year (every 3 months or more)**	0	0	2	29	11	92	8	80	4	80	4	80	0	0
	**3 times/year (every 4 months)**	7	100	4	57	0	0	1	10	0	0	1	20	0	0
	**2 times/year (every 6 months)**	0	0	1	14	1	8	1	10	0	0	0	0	1	100
	**Once a year**	0	0	0	0	0	0	0	0	0	0	0	0	0	0
	**No regular follow-up**	0	0	0	0	0	0	0	0	1	20	0	0	0	0
**Research Capabilities**															

**Q27: What percentage of research staff have GCP certification?**	**>75% staff certified**	2	29	4	57	0	0	2	20	5	100	0	0	0	0
	**50-75% staff certified**	3	43	0	0	1	8	2	20	0	0	0	0	0	0
	**25-50% staff certified**	2	29	1	14	5	42	4	40	0	0	0	0	0	0
	**<25% staff certified**	0	0	2	29	6	50	2	20	0	0	5	100	1	100
**Q28: Do staff have experience in clinical research studies?**	**>75% staff experienced**	1	14	4	57	1	8	1	10	4	80	0	0	1	100
	**50-75% staff experienced**	2	29	1	14	1	8	3	30	1	20	1	20	0	0
	**25-50% staff experienced**	4	57	0	0	4	33	2	20	0	0	1	20	0	0
	**<25% staff experienced**	0	0	2	29	6	50	4	40	0	0	3	60	0	0
**Q29: Are there established guidelines for handling digital patient data?**	**Guidelines exist, followed >75%**	1	14	6	86	2	17	1	10	5	100	1	20	0	0
	**Guidelines exist, followed 50-75%**	4	57	0	0	1	8	2	20	0	0	0	0	1	100
	**Guidelines exist, followed <50%**	2	29	0	0	3	25	1	10	0	0	1	20	0	0
	**No guidelines**	0	0	1	14	6	50	6	60	0	0	3	60	0	0
**Q30: Are staff trained in data privacy in digital environment?**	**>75% staff trained**	2	29	5	71	1	8	4	40	5	100	2	40	1	100
	**50-75% staff trained**	3	43	1	14	1	8	2	20	0	0	1	20	0	0
	**25-50% staff trained**	2	29	0	0	2	17	0	0	0	0	0	0	0	0
	**<25% staff trained**	0	0	1	14	5	42	1	10	0	0	0	0	0	0
	**Not trained**	0	0	0	0	3	25	3	30	0	0	2	40	0	0
**Q31: Do you have an IRB/Ethics Committee locally?**	**Local IRB**	1	14	4	57	1	8	4	40	3	60	3	60	1	100

	**Affiliate IRB**	0	0	3	43	3	25	3	30	2	40	1	20	0	0
	**No IRB available**	6	86	0	0	8	67	3	30	0	0	1	20	0	0
**Q32: How frequently does your IRB/Ethics Committee meet?**	**Weekly**	0	0	0	0	0	0	0	0	0	0	0	0	0	0
	**Monthly**	0	0	5	71	1	8	1	10	3	60	2	40	1	100
	**Every 2-3 months**	0	0	0	0	1	8	5	50	0	0	0	0	0	0
	**Less frequently than every 3 months**	1	14	2	29	1	8	2	20	2	40	1	20	0	0
	**No IRB**	6	86	0	0	9	75	2	20	0	0	2	40	0	0
**Q33: Are there quality assurance procedures for data collection?**	**Procedures exist and followed**	7	100	5	71	4	33	4	40	5	100	0	0	0	0
	**Procedures exist but not followed**	0	0	1	14	2	17	3	30	0	0	0	0	1	100
	**No procedures**	0	0	1	14	6	50	3	30	0	0	5	100	0	0
**Finance and Administration**															
**Q34: Have you previously acquired grants/funding for research?**	**Yes**	2	29	4	57	3	25	3	30	3	60	2	40	0	0
	**No**	5	71	3	43	9	75	7	70	2	40	3	60	1	100
**Q35: How much time can admin staff allocate to research support (average hours per staff)?**	**<4 hours/week per staff**	3	43	5	71	5	42	6	60	3	60	4	80	0	0
	**4-8 hours/week per staff**	3	43	1	14	3	25	2	20	2	40	1	20	0	0
	**8-12 hours/week per staff**	1	14	0	0	1	8	1	10	0	0	0	0	1	100
	**12-16 hours/week per staff**	0	0	0	0	0	0	1	10	0	0	0	0	0	0
	**>16 hours/week per staff**	0	0	1	14	2	17	0	0	0	0	0	0	0	0

**Collaboration and Networking**															
**Q36: Does the clinic participate in diabetes research networks?**	**National + international networks**	1	14	4	57	3	25	2	20	4	80	0	0	0	0
	**National networks only**	6	86	3	43	5	42	5	50	0	0	2	40	1	100
	**Does not participate**	0	0	0	0	4	33	3	30	1	20	3	60	0	0

Supplementary Table S4: Question Results by Country (ETH, GHA, GIN, IND, IDN, KEN, MYS)

Question	Component	ETH		GHA		GIN		IND		IDN		KEN		MYS	
**Clinical Staffing and Experience**		(n = 32)	%	(n = 1)	%	(n = 8)	%	(n = 51)	%	(n = 12)	%	(n = 28)	%	(n = 33)	%
**Q1: Please indicate which of the below practitioners are available at your centre to treat T1D in patients ≤20 years old and how many**	**Nurses**	32	100	0	0	6	75	23	45	9	75	25	89	13	39
	**Endocrinologists/Diabetologists**	2	6	1	100	3	38	44	86	8	67	7	25	26	79
	**Dietician**	0	0	1	100	0	0	44	86	10	83	21	75	27	82
	**General physician/non-specialist**	29	91	0	0	5	62	17	33	4	33	21	75	13	39
	**Paediatrician (non-endo)**	30	94	1	100	0	0	19	37	7	58	21	75	18	55
	**Specialist nurses**	1	3	1	100	5	62	20	39	7	58	8	29	24	73
	**Psychologist/counsellor**	0	0	1	100	0	0	24	47	4	33	25	89	12	36
	**Medical assistants**	0	0	0	0	0	0	36	71	3	25	17	61	17	52
	**Paediatric endocrinologist**	2	6	1	100	1	12	21	41	12	100	8	29	11	33
	**Social worker**	0	0	0	0	0	0	20	39	2	17	21	75	12	36
	**Trainee physician**	1	3	0	0	3	38	19	37	3	25	12	43	5	15
**Health Services**															
**Q2: Please indicate which services to screen for T1D complications are routinely provided**	**Clinical Exam**	32	100	1	100	7	88	51	100	12	100	28	100	32	97
	**Eye Fundus Exam**	22	69	1	100	6	75	42	82	11	92	18	64	28	85
	**Foot Exam**	32	100	1	100	4	50	46	90	5	42	22	79	26	79
	**Height and weight measurement**	32	100	1	100	5	62	51	100	12	100	28	100	31	94
**Q3: What equipment do most (>50%) children use to check blood glucose?**	**Glucometer + test strips**	32	100	1	100	8	100	49	96	12	100	28	100	32	97
	**Continuous Glucose Monitoring Device**	0	0	0	0	0	0	2	4	0	0	0	0	1	3

	**None**	0	0	0	0	0	0	0	0	0	0	0	0	0	0
**Q4: What equipment do you provide for blood glucose/ketone monitoring (free or paid)?**	**For Free: Glucometer**	32	100	1	100	8	100	33	65	3	25	24	86	19	58
	**For Free: Test strips**	32	100	1	100	7	88	30	59	3	25	25	89	20	61
	**For Free: Urine ketone strips**	0	0	1	100	1	12	6	12	0	0	6	21	1	3
	**For Free: Capillary ketone monitor**	0	0	0	0	0	0	0	0	0	0	0	0	2	6
	**For Free: CGM**	0	0	0	0	1	12	2	4	0	0	3	11	3	9
	**Paid: Glucometer**	0	0	0	0	2	25	22	43	7	58	6	21	13	39
	**Paid: Test strips**	0	0	0	0	2	25	27	53	6	50	4	14	14	42
	**Paid: CGM**	0	0	0	0	0	0	24	47	1	8	0	0	21	64
	**Paid: Capillary Ketone Monitor**	0	0	0	0	0	0	8	16	4	33	1	4	10	30
	**Paid: Urine Ketone Strips**	0	0	0	0	4	50	19	37	3	25	9	32	3	9
	**None**	0	0	0	0	0	0	2	4	3	17	0	0	7	21
**Q5: What insulin delivery do most (>50%) children use?**	**Subcutaneous Insulin Syringe**	32	100	1	100	8	100	10	20	0	0	0	0	1	3
	**Subcutaneous Insulin Pen**	0	0	0	0	0	0	40	78	12	100	28	100	32	97
	**Insulin Pump**	0	0	0	0	0	0	1	2	0	0	0	0	0	0
**Q6: Is acute DKA managed at your facility? (from diagnosis to discharge)**	**No**	0	0	0	0	0	0	7	14	0	0	0	0	0	0
	**Only diagnosis**	0	0	0	0	0	0	17	33	0	0	3	11	0	0
	**Yes - From diagnosis to treatment to discharge**	32	100	1	100	8	100	27	53	12	100	25	89	33	100
**Q7: Which of the following does your clinic have to treat acute DKA? (% of**	**None**	1	3	0	0	0	0	5	11	0	0	0	0	0	0

**those not answering no to Q6)**															
	**Capillary glucose monitor**	31	97	1	100	7	88	46	105	10	83	21	75	32	97
	**Capillary ketone monitor**	0	0	0	0	1	12	18	41	5	42	6	21	32	97
	**POC blood gas analysis**	1	3	0	0	0	0	27	61	12	100	10	36	30	91
	**Continuous cardiac monitoring/pulse oximetry**	30	94	0	0	0	0	32	73	12	100	20	71	28	85
	**Fast-acting insulin**	30	94	1	100	6	75	42	95	12	100	27	96	30	91
	**IV fluids**	31	97	1	100	5	62	38	86	12	100	27	96	33	100
	**Potassium replacement**	31	97	1	100	7	88	33	75	12	100	24	86	33	100
**Q8: What proportion receive structured diabetes education at diagnosis?**	**0-25%**	20	62	0	0	0	0	1	2	1	8	1	4	2	6
	**25-50%**	12	38	0	0	0	0	6	12	0	0	1	4	6	18
	**50-75%**	0	0	0	0	3	38	5	10	3	25	8	29	6	18
	**75-100%**	0	0	1	100	5	62	39	76	8	67	18	64	19	58
**Q9: What proportion receive services without paying at point of care?**	**None**	0	0	0	0	1	13	4	8	0	0	6	21	1	3
	**<25%**	4	12	0	0	0	0	10	20	0	0	3	11	1	3
	**25-50%**	14	44	0	0	2	25	10	20	1	8	0	0	5	15
	**50-75%**	12	38	0	0	0	0	6	12	2	17	4	14	7	21
	**75-<100%**	2	6	1	100	3	38	7	14	3	25	9	32	9	27
	**100%**	0	0	0	0	2	25	14	27	6	50	6	21	10	30
**Q10: Are there private spaces for patient consultations?**	**Yes**	11	34	0	0	6	75	49	96	9	75	22	79	26	79
	**No**	21	66	1	100	2	25	2	4	3	25	6	21	7	21

**Q11: Is telehealth capability available?**	**Yes**	1	3	0	0	0	0	41	80	6	50	7	25	10	30
	**No**	31	97	1	100	8	100	10	20	6	50	21	75	23	70
**Resource Management**															
**Q12: What digital devices are in place to manage patient data?**	**Both computers and mobile devices**	0	0	1	100	0	0	39	76	6	50	13	46	10	30
	**Either computers or mobile devices**	5	16	0	0	0	0	10	20	6	50	10	36	18	55
	**No computer or mobile device**	27	84	0	0	8	100	2	4	0	0	5	18	5	15
**Q13: Are software programs available and up-to-date for digital patient information?**	**Software available and up-to date**	2	6	0	0	0	0	33	65	1	8	7	25	7	21
	**Software available but not up-to-date**	0	0	1	100	2	25	10	20	6	50	14	50	5	15
	**No software available**	30	94	0	0	6	75	8	16	5	42	7	25	21	64
**Q14: How often does your facility experience insulin stock outs?**	**Stock outs 0-5% (0-19 days)**	2	6	0	0	7	88	36	71	6	50	12	43	19	58
	**Stock outs 5-10% (20-40 days)**	22	69	0	0	0	0	3	6	1	8	10	36	6	18
	**Stock outs 10-15% (41-60 days)**	6	19	0	0	0	0	2	4	1	8	0	0	2	6
	**Stock outs 15-20% (61-80 days)**	0	0	0	0	0	0	1	2	1	8	3	11	0	0
	**Stock outs 20-30% (81-120 days)**	0	0	0	0	0	0	5	10	0	0	1	4	4	12
	**Stock outs >30% of year (>120 days)**	0	0	1	100	0	0	0	0	3	25	2	7	2	6
	**Insulin not stocked/provided**	2	6	0	0	1	12	4	8	0	0	0	0	0	0
**Q15: How often does your facility experience glucose equipment stock outs?**	**Stock outs 0-5% (0-19 days)**	1	3	0	0	1	12	33	65	3	25	5	18	23	70

	Stock outs 5-10% (20-40 days)	20	62	0	0	1	12	6	12	0	0	4	14	2	6
	Stock outs 10-15% (41-60 days)	10	31	0	0	0	0	0	0	0	0	5	18	0	0
	Stock outs 15-20% (61-80 days)	1	3	0	0	0	0	1	2	1	8	1	4	0	0
	Stock outs 20-30% (81-120 days)	0	0	0	0	1	12	3	6	1	8	4	14	0	0
	Stock outs >30% of year (>120 days)	0	0	1	100	5	62	3	6	2	17	7	25	4	12
	Equipment not stocked/provided	0	0	0	0	0	0	5	10	5	42	2	7	4	12
Q16: Does your facility have software for inventory management?	Software-based inventory management	0	0	0	0	0	0	15	29	4	33	8	29	6	18
	Manual inventory management	32	100	1	100	3	38	27	53	2	17	19	68	18	55
	No inventory management	0	0	0	0	5	62	9	18	6	50	1	4	9	27
Q17: Is the clinic equipped with stable internet connectivity?	Internet >75% of time	7	22	0	0	0	0	49	96	9	75	12	43	17	52
	Internet 50-75% of time	0	0	0	0	1	12	1	2	1	8	4	14	7	21
	Intermittent internet	0	0	0	0	0	0	1	2	1	8	4	14	5	15
	No internet	25	78	1	100	7	88	0	0	1	8	8	29	4	12
Testing Facilities															
Q18: Which lab tests are available for T1D patients?	HbA1c	32	100	1	100	8	100	50	98	12	100	27	96	33	100
	Lipid profile	17	53	0	0	5	62	49	96	12	100	17	61	32	97
	Serum creatinine	25	78	0	0	5	62	47	92	12	100	19	68	33	100
	Urine creatinine	3	9	0	0	3	38	41	80	9	75	8	29	25	76
	Thyroid function test	16	50	0	0	0	0	46	90	7	58	16	57	33	100
	Full blood count	26	81	0	0	5	62	46	90	12	100	21	75	33	100

	**None**	0	0	0	0	0	0	1	2	0	0	0	0	0	0
**Q19: Which tests are available/accessed?**	**Oral glucose tolerance testing**	7	22	0	0	3	38	44	86	5	42	16	57	33	100
	**Pancreatic autoantibody**	0	0	0	0	0	0	37	73	1	8	1	4	28	85
	**C-peptide testing**	0	0	0	0	0	0	44	86	6	50	3	11	30	91
**Q20: Does the facility have access to genetic testing for T1D patients?**	**Yes**	1	3	0	0	0	0	24	47	1	8	0	0	7	21
	**No**	31	97	1	100	8	100	27	53	11	92	28	100	26	79
**Accessibility**															
**Q21: What proportion of T1D patients travel over 60 minutes to reach your centre?**	**66-100%**	1	3	0	0	3	38	15	29	5	42	8	29	2	6
	**33-66%**	25	78	0	0	5	62	29	57	4	33	18	64	11	33
	**<33%**	6	19	1	100	0	0	7	14	2	17	2	7	18	55
	**None**	0	0	0	0	0	0	0	0	1	8	0	0	2	6
**Q22: Is the clinic equipped for language support for diverse populations?**	**Support for multiple languages**	24	75	1	100	0	0	42	82	3	25	14	50	21	64
	**Support for one local language**	7	22	0	0	1	12	7	14	3	25	9	32	5	15
	**Not equipped**	1	3	0	0	7	88	2	4	6	50	5	18	7	21
**Data Management**															
**Q23: Is the clinic adequately staffed for patient data recording for research?**	**>75% of required staff**	29	91	0	0	0	0	26	51	4	33	6	21	2	6

	**25-75% of required staff**	0	0	0	0	1	12	19	37	0	0	16	57	13	39
	**<25% of required staff**	3	9	1	100	7	88	6	12	8	67	6	21	18	55
**Q24: Can systems integrate electronic patient records from external health centers?**	**Full integration (75-100%)**	0	0	0	0	0	0	9	18	2	17	4	14	2	6
	**Majority integration (25-75%)**	0	0	0	0	0	0	7	14	2	17	2	7	2	6
	**Some integration (<25%)**	0	0	0	0	1	12	4	8	3	25	2	7	2	6
	**No integration (0%)**	4	12	1	100	1	12	23	45	5	42	11	39	14	42
	**No digital systems**	28	88	0	0	6	75	8	16	0	0	9	32	13	39
**Q25: Does the clinic have ability to store study data securely?**	**>75% securely stored**	3	9	0	0	1	12	30	59	6	50	16	57	8	24
	**50-75% securely stored**	0	0	0	0	0	0	5	10	0	0	2	7	5	15
	**<50% securely stored**	0	0	1	100	1	12	5	10	3	25	6	21	2	6
	**Ability but done 0%**	1	3	0	0	2	25	7	14	2	17	0	0	3	9
	**No ability**	28	88	0	0	4	50	4	8	1	8	4	14	15	45
**Patient Engagement**															
**Q26: Does the clinic conduct regular follow-up with T1D patients?**	**≥4 times/year (every 3 months or more)**	32	100	1	100	8	100	35	69	7	58	23	82	22	67
	**3 times/year (every 4 months)**	0	0	0	0	0	0	12	24	1	8	1	4	9	27
	**2 times/year (every 6 months)**	0	0	0	0	0	0	2	4	1	8	0	0	2	6
	**Once a year**	0	0	0	0	0	0	2	4	1	8	3	11	0	0
	**No regular follow-up**	0	0	0	0	0	0	0	0	2	17	1	4	0	0
**Research Capabilities**															

**Q27: What percentage of research staff have GCP certification?**	**>75% staff certified**	2	6	0	0	1	12	12	24	4	33	8	29	7	21
	**50-75% staff certified**	0	0	1	100	0	0	5	10	1	8	5	18	6	18
	**25-50% staff certified**	0	0	0	0	0	0	10	20	2	17	5	18	5	15
	**<25% staff certified**	30	94	0	0	7	88	24	47	5	42	10	36	15	45
**Q28: Do staff have experience in clinical research studies?**	**>75% staff experienced**	0	0	1	100	0	0	9	18	3	25	6	21	2	6
	**50-75% staff experienced**	0	0	0	0	0	0	8	16	2	17	4	14	3	9
	**25-50% staff experienced**	0	0	0	0	0	0	16	31	0	0	5	18	13	39
	**<25% staff experienced**	32	100	0	0	8	100	18	35	7	58	13	46	15	45
**Q29: Are there established guidelines for handling digital patient data?**	**Guidelines exist, followed >75%**	0	0	1	100	1	12	24	47	3	25	13	46	6	18
	**Guidelines exist, followed 50-75%**	1	3	0	0	0	0	6	12	1	8	5	18	3	9
	**Guidelines exist, followed <50%**	2	6	0	0	3	38	5	10	2	17	5	18	6	18
	**No guidelines**	29	91	0	0	4	50	16	31	6	50	5	18	18	55
**Q30: Are staff trained in data privacy in digital environment?**	**>75% staff trained**	0	0	0	0	2	25	22	43	3	25	10	36	6	18
	**50-75% staff trained**	0	0	1	100	0	0	4	8	1	8	5	18	4	12
	**25-50% staff trained**	1	3	0	0	0	0	5	10	1	8	3	11	4	12
	**<25% staff trained**	1	3	0	0	2	25	14	27	3	25	6	21	8	24
	**Not trained**	30	94	0	0	4	50	6	12	4	33	4	14	11	33
**Q31: Do you have an IRB/Ethics Committee locally?**	**Local IRB**	6	19	1	100	0	0	25	49	11	92	10	36	15	45

	**Affiliate IRB**	0	0	0	0	0	0	15	29	0	0	10	36	12	36
	**No IRB available**	26	81	0	0	8	100	11	22	1	8	8	29	6	18
**Q32: How frequently does your IRB/Ethics Committee meet?**	**Weekly**	0	0	0	0	0	0	0	0	0	0	0	0	0	0
	**Monthly**	6	19	0	0	0	0	16	31	5	42	3	11	5	15
	**Every 2-3 months**	0	0	1	100	0	0	15	29	0	0	10	36	7	21
	**Less frequently than every 3 months**	0	0	0	0	1	12	7	14	6	50	5	18	11	33
	**No IRB**	26	81	0	0	7	88	13	25	1	8	10	36	10	30
**Q33: Are there quality assurance procedures for data collection?**	**Procedures exist and followed**	1	3	1	100	0	0	31	61	6	50	15	54	17	52
	**Procedures exist but not followed**	8	25	0	0	0	0	5	10	4	33	7	25	4	12
	**No procedures**	23	72	0	0	8	100	15	29	2	17	6	21	12	36
**Finance and Administration**															
**Q34: Have you previously acquired grants/funding for research?**	**Yes**	3	9	1	100	0	0	26	51	3	25	7	25	12	36
	**No**	29	91	0	0	8	100	25	49	9	75	21	75	21	64
**Q35: How much time can admin staff allocate to research support (average hours per staff)?**	**<4 hours/week per staff**	32	100	0	0	6	75	16	31	5	42	8	29	22	67
	**4-8 hours/week per staff**	0	0	0	0	1	12	11	22	2	17	7	25	5	15
	**8-12 hours/week per staff**	0	0	1	100	0	0	8	16	0	0	5	18	3	9
	**12-16 hours/week per staff**	0	0	0	0	0	0	2	4	0	0	4	14	1	3
	**>16 hours/week per staff**	0	0	0	0	0	0	14	27	5	42	2	7	1	3

**Collaboration and Networking**															
**Q36: Does the clinic participate in diabetes research networks?**	**National + international networks**	1	3	1	100	0	0	21	41	4	33	12	43	8	24
	**National networks only**	1	3	0	0	2	25	11	22	5	42	8	29	11	33
	**Does not participate**	30	94	0	0	6	75	19	37	3	25	8	29	14	42

Supplementary Table S5: Question Results by Country (MAR, NER, NGA, PAK, PER, SEN, VNM)

Question	Component	MAR		NER		NGA		PAK		PER		SEN		VNM	
**Clinical Staffing and Experience**		(n = 1)	%	(n = 1)	%	(n = 1)	%	(n = 24)	%	(n = 3)	%	(n = 1)	%	(n = 1)	%
**Q1: Please indicate which of the below practitioners are available at your centre to treat T1D in patients ≤20 years old and how many**	**Nurses**	0	0	1	100	1	100	1	4	3	100	1	100	1	100
	**Endocrinologists/Diabetologists**	0	0	1	100	0	0	24	100	3	100	1	100	0	0
	**Dietician**	1	100	1	100	1	100	24	100	3	100	1	100	0	0
	**General physician/non-specialist**	0	0	1	100	0	0	1	4	1	33	0	0	0	0
	**Paediatrician (non-endo)**	0	0	1	100	0	0	1	4	2	67	1	100	0	0
	**Specialist nurses**	1	100	1	100	0	0	24	100	1	33	0	0	0	0
	**Psychologist/counsellor**	1	100	1	100	0	0	1	4	3	100	0	0	0	0
	**Medical assistants**	0	0	1	100	0	0	1	4	2	67	0	0	0	0
	**Paediatric endocrinologist**	1	100	1	100	1	100	1	4	2	67	1	100	1	100
	**Social worker**	1	100	1	100	0	0	0	0	2	67	0	0	0	0
	**Trainee physician**	0	0	1	100	1	100	1	4	0	0	1	100	1	100
**Health Services**															
**Q2: Please indicate which services to screen for T1D complications are routinely provided**	**Clinical Exam**	1	100	1	100	1	100	24	100	3	100	1	100	1	100
	**Eye Fundus Exam**	0	0	0	0	0	0	24	100	3	100	1	100	1	100
	**Foot Exam**	1	100	1	100	0	0	24	100	2	67	1	100	0	0
	**Height and weight measurement**	1	100	1	100	1	100	24	100	3	100	1	100	1	100
**Q3: What equipment do most (>50%) children use to check blood glucose?**	**Glucometer + test strips**	1	100	1	100	1	100	24	100	3	100	1	100	1	100
	**Continuous Glucose Monitoring Device**	0	0	0	0	0	0	0	0	0	0	0	0	0	0
	**None**	0	0	0	0	0	0	0	0	0	0	0	0	0	0

**Q4: What equipment do you provide for blood glucose/ketone monitoring (free or paid)?**	**For Free: Glucometer**	1	100	0	0	0	0	24	100	2	67	1	100	0	0
	**For Free: Test strips**	1	100	0	0	0	0	24	100	3	100	1	100	0	0
	**For Free: Urine ketone strips**	1	100	0	0	0	0	0	0	0	0	0	0	0	0
	**For Free: Capillary ketone monitor**	1	100	0	0	0	0	0	0	0	0	0	0	0	0
	**For Free: CGM**	0	0	0	0	0	0	0	0	0	0	0	0	0	0
	**Paid: Glucometer**	0	0	1	100	1	100	0	0	0	0	0	0	0	0
	**Paid: Test strips**	0	0	1	100	1	100	0	0	0	0	0	0	0	0
	**Paid: CGM**	1	100	0	0	0	0	0	0	0	0	1	100	0	0
	**Paid: Capillary Ketone Monitor**	0	0	0	0	0	0	0	0	0	0	1	100	0	0
	**Paid: Urine Ketone Strips**	0	0	1	100	1	100	0	0	0	0	1	100	0	0
	**None**	0	0	0	0	0	0	0	0	0	0	0	0	1	100
**Q5: What insulin delivery do most (>50%) children use?**	**Subcutaneous Insulin Syringe**	0	0	1	100	0	0	0	0	1	33	1	100	1	100
	**Subcutaneous Insulin Pen**	1	100	0	0	1	100	24	100	2	67	0	0	0	0
	**Insulin Pump**	0	0	0	0	0	0	0	0	0	0	0	0	0	0
**Q6: Is acute DKA managed at your facility? (from diagnosis to discharge)**	**No**	0	0	0	0	0	0	0	0	0	0	0	0	0	0
	**Only diagnosis**	0	0	0	0	0	0	22	92	0	0	0	0	0	0
	**Yes - From diagnosis to treatment to discharge**	1	100	1	100	1	100	2	8	3	100	1	100	1	100
**Q7: Which of the following does your clinic have to treat acute DKA? (% of those not answering no to Q6)**	**None**	0	0	0	0	0	0	0	0	0	0	0	0	0	0

	**Capillary glucose monitor**	1	100	1	100	1	100	0	0	2	67	1	100	1	100
	**Capillary ketone monitor**	1	100	0	0	0	0	1	4	1	33	0	0	1	100
	**POC blood gas analysis**	1	100	0	0	0	0	1	4	2	67	0	0	1	100
	**Continuous cardiac monitoring/pulse oximetry**	1	100	0	0	1	100	1	4	2	67	1	100	1	100
	**Fast-acting insulin**	1	100	1	100	1	100	24	100	3	100	1	100	1	100
	**IV fluids**	1	100	1	100	1	100	24	100	3	100	1	100	1	100
	**Potassium replacement**	1	100	1	100	1	100	1	4	1	33	1	100	1	100
**Q8: What proportion receive structured diabetes education at diagnosis?**	**0-25%**	0	0	0	0	0	0	0	0	1	33	0	0	0	0
	**25-50%**	0	0	0	0	0	0	0	0	1	33	0	0	0	0
	**50-75%**	0	0	1	100	0	0	1	4	0	0	0	0	0	0
	**75-100%**	1	100	0	0	1	100	23	96	1	33	1	100	1	100
**Q9: What proportion receive services without paying at point of care?**	**None**	0	0	0	0	1	100	0	0	0	0	1	100	0	0
	**<25%**	0	0	1	100	0	0	0	0	0	0	0	0	0	0
	**25-50%**	0	0	0	0	0	0	0	0	0	0	0	0	0	0
	**50-75%**	1	100	0	0	0	0	0	0	0	0	0	0	1	100
	**75-<100%**	0	0	0	0	0	0	1	4	1	33	0	0	0	0
	**100%**	0	0	0	0	0	0	23	96	2	67	0	0	0	0
**Q10: Are there private spaces for patient consultations?**	**Yes**	1	100	1	100	1	100	24	100	2	67	0	0	1	100
	**No**	0	0	0	0	0	0	0	0	1	33	1	100	0	0
**Q11: Is telehealth capability available?**	**Yes**	1	100	1	100	0	0	24	100	3	100	1	100	0	0

	**No**	0	0	0	0	1	100	0	0	0	0	0	0	1	100
**Resource Management**															
**Q12: What digital devices are in place to manage patient data?**	**Both computers and mobile devices**	1	100	0	0	0	0	24	100	1	33	0	0	0	0
	**Either computers or mobile devices**	0	0	1	100	1	100	0	0	1	33	0	0	1	100
	**No computer or mobile device**	0	0	0	0	0	0	0	0	1	33	1	100	0	0
**Q13: Are software programs available and up-to-date for digital patient information?**	**Software available and up-to date**	1	100	0	0	0	0	24	100	1	33	0	0	1	100
	**Software available but not up-to-date**	0	0	0	0	1	100	0	0	0	0	1	100	0	0
	**No software available**	0	0	1	100	0	0	0	0	2	67	0	0	0	0
**Q14: How often does your facility experience insulin stock outs?**	**Stock outs 0-5% (0-19 days)**	0	0	1	100	1	100	24	100	3	100	0	0	1	100
	**Stock outs 5-10% (20-40 days)**	0	0	0	0	0	0	0	0	0	0	1	100	0	0
	**Stock outs 10-15% (41-60 days)**	0	0	0	0	0	0	0	0	0	0	0	0	0	0
	**Stock outs 15-20% (61-80 days)**	0	0	0	0	0	0	0	0	0	0	0	0	0	0
	**Stock outs 20-30% (81-120 days)**	1	100	0	0	0	0	0	0	0	0	0	0	0	0
	**Stock outs >30% of year (>120 days)**	0	0	0	0	0	0	0	0	0	0	0	0	0	0
	**Insulin not stocked/provided**	0	0	0	0	0	0	0	0	0	0	0	0	0	0
**Q15: How often does your facility experience glucose equipment stock outs?**	**Stock outs 0-5% (0-19 days)**	0	0	0	0	0	0	24	100	3	100	0	0	0	0
	**Stock outs 5-10% (20-40 days)**	0	0	0	0	0	0	0	0	0	0	0	0	0	0
	**Stock outs 10-15% (41-60 days)**	0	0	0	0	0	0	0	0	0	0	1	100	0	0
	**Stock outs 15-20% (61-80 days)**	0	0	0	0	0	0	0	0	0	0	0	0	0	0

	**Stock outs 20-30% (81-120 days)**	0	0	0	0	0	0	0	0	0	0	0	0	0	0
	**Stock outs >30% of year (>120 days)**	1	100	0	0	0	0	0	0	0	0	0	0	0	0
	**Equipment not stocked/provided**	0	0	1	100	1	100	0	0	0	0	0	0	1	100
**Q16: Does your facility have software for inventory management?**	**Software-based inventory management**	0	0	0	0	0	0	0	0	1	33	0	0	0	0
	**Manual inventory management**	1	100	1	100	1	100	24	100	1	33	1	100	0	0
	**No inventory management**	0	0	0	0	0	0	0	0	1	33	0	0	1	100
**Q17: Is the clinic equipped with stable internet connectivity?**	**Internet >75% of time**	1	100	0	0	0	0	0	0	2	67	0	0	0	0
	**Internet 50-75% of time**	0	0	0	0	1	100	0	0	1	33	1	100	1	100
	**Intermittent internet**	0	0	1	100	0	0	24	100	0	0	0	0	0	0
	**No internet**	0	0	0	0	0	0	0	0	0	0	0	0	0	0
**Testing Facilities**															
**Q18: Which lab tests are available for T1D patients?**	**HbA1c**	1	100	0	0	1	100	24	100	3	100	1	100	1	100
	**Lipid profile**	0	0	0	0	1	100	0	0	3	100	0	0	1	100
	**Serum creatinine**	0	0	0	0	1	100	0	0	3	100	0	0	1	100
	**Urine creatinine**	0	0	0	0	0	0	0	0	2	67	0	0	1	100
	**Thyroid function test**	0	0	0	0	1	100	0	0	1	33	0	0	1	100
	**Full blood count**	0	0	0	0	1	100	0	0	3	100	0	0	1	100
	**None**	0	0	1	100	0	0	0	0	0	0	0	0	0	0
**Q19: Which tests are available/accessed?**	**Oral glucose tolerance testing**	0	0	0	0	0	0	2	8	3	100	1	100	1	100
	**Pancreatic autoantibody**	1	100	0	0	1	100	2	8	0	0	0	0	1	100

	**C-peptide testing**	1	100	0	0	1	100	2	8	1	33	0	0	1	100
**Q20: Does the facility have access to genetic testing for T1D patients?**	**Yes**	0	0	0	0	0	0	0	0	0	0	0	0	1	100
	**No**	1	100	1	100	1	100	24	100	3	100	1	100	0	0
**Accessibility**															
**Q21: What proportion of T1D patients travel over 60 minutes to reach your centre?**	**66-100%**	0	0	1	100	1	100	0	0	2	67	0	0	1	100
	**33-66%**	0	0	0	0	0	0	24	100	0	0	1	100	0	0
	**<33%**	1	100	0	0	0	0	0	0	1	33	0	0	0	0
	**None**	0	0	0	0	0	0	0	0	0	0	0	0	0	0
**Q22: Is the clinic equipped for language support for diverse populations?**	**Support for multiple languages**	0	0	0	0	1	100	24	100	0	0	0	0	0	0
	**Support for one local language**	1	100	0	0	0	0	0	0	2	67	0	0	0	0
	**Not equipped**	0	0	1	100	0	0	0	0	1	33	1	100	1	100
**Data Management**															
**Q23: Is the clinic adequately staffed for patient data recording for research?**	**>75% of required staff**	0	0	0	0	1	100	1	4	1	33	0	0	1	100
	**25-75% of required staff**	0	0	0	0	0	0	23	96	0	0	0	0	0	0
	**<25% of required staff**	1	100	1	100	0	0	0	0	2	67	1	100	0	0
**Q24: Can systems integrate electronic patient records from external health centers?**	**Full integration (75-100%)**	0	0	0	0	0	0	24	100	0	0	0	0	0	0

	**Majority integration (25-75%)**	0	0	0	0	0	0	0	0	0	0	0	0	0	0
	**Some integration (<25%)**	1	100	0	0	0	0	0	0	0	0	0	0	0	0
	**No integration (0%)**	0	0	0	0	0	0	0	0	2	67	1	100	0	0
	**No digital systems**	0	0	1	100	1	100	0	0	1	33	0	0	1	100
**Q25: Does the clinic have ability to store study data securely?**	**>75% securely stored**	1	100	0	0	0	0	24	100	1	33	0	0	1	100
	**50-75% securely stored**	0	0	0	0	0	0	0	0	0	0	0	0	0	0
	**<50% securely stored**	0	0	1	100	0	0	0	0	1	33	0	0	0	0
	**Ability but done 0%**	0	0	0	0	1	100	0	0	1	33	1	100	0	0
	**No ability**	0	0	0	0	0	0	0	0	0	0	0	0	0	0
**Patient Engagement**															
**Q26: Does the clinic conduct regular follow-up with T1D patients?**	**≥4 times/year (every 3 months or more)**	0	0	1	100	1	100	24	100	2	67	0	0	1	100
	**3 times/year (every 4 months)**	1	100	0	0	0	0	0	0	0	0	0	0	0	0
	**2 times/year (every 6 months)**	0	0	0	0	0	0	0	0	1	33	0	0	0	0
	**Once a year**	0	0	0	0	0	0	0	0	0	0	1	100	0	0
	**No regular follow-up**	0	0	0	0	0	0	0	0	0	0	0	0	0	0
**Research Capabilities**															
**Q27: What percentage of research staff have GCP certification?**	**>75% staff certified**	0	0	0	0	0	0	0	0	0	0	0	0	1	100
	**50-75% staff certified**	0	0	0	0	0	0	23	96	1	33	0	0	0	0
	**25-50% staff certified**	0	0	1	100	0	0	1	4	0	0	0	0	0	0
	**<25% staff certified**	1	100	0	0	1	100	0	0	2	67	1	100	0	0

**Q28: Do staff have experience in clinical research studies?**	**>75% staff experienced**	0	0	1	100	1	100	0	0	0	0	0	0	0	0
	**50-75% staff experienced**	0	0	0	0	0	0	0	0	1	33	0	0	1	100
	**25-50% staff experienced**	1	100	0	0	0	0	0	0	1	33	0	0	0	0
	**<25% staff experienced**	0	0	0	0	0	0	24	100	1	33	1	100	0	0
**Q29: Are there established guidelines for handling digital patient data?**	**Guidelines exist, followed >75%**	1	100	1	100	0	0	0	0	2	67	0	0	1	100
	**Guidelines exist, followed 50-75%**	0	0	0	0	0	0	24	100	0	0	0	0	0	0
	**Guidelines exist, followed <50%**	0	0	0	0	0	0	0	0	0	0	0	0	0	0
	**No guidelines**	0	0	0	0	1	100	0	0	1	33	1	100	0	0
**Q30: Are staff trained in data privacy in digital environment?**	**>75% staff trained**	1	100	0	0	0	0	24	100	1	33	0	0	1	100
	**50-75% staff trained**	0	0	0	0	0	0	0	0	1	33	0	0	0	0
	**25-50% staff trained**	0	0	1	100	0	0	0	0	0	0	0	0	0	0
	**<25% staff trained**	0	0	0	0	0	0	0	0	0	0	0	0	0	0
	**Not trained**	0	0	0	0	1	100	0	0	1	33	1	100	0	0
**Q31: Do you have an IRB/Ethics Committee locally?**	**Local IRB**	1	100	0	0	1	100	0	0	2	67	0	0	0	0
	**Affiliate IRB**	0	0	1	100	0	0	0	0	1	33	1	100	1	100
	**No IRB available**	0	0	0	0	0	0	24	100	0	0	0	0	0	0
**Q32: How frequently does your IRB/Ethics Committee meet?**	**Weekly**	0	0	0	0	0	0	0	0	0	0	0	0	0	0
	**Monthly**	0	0	1	100	1	100	0	0	1	33	0	0	1	100
	**Every 2-3 months**	0	0	0	0	0	0	0	0	0	0	0	0	0	0

	**Less frequently than every 3 months**	1	100	0	0	0	0	0	0	1	33	0	0	0	0
	**No IRB**	0	0	0	0	0	0	24	100	1	33	1	100	0	0
**Q33: Are there quality assurance procedures for data collection?**	**Procedures exist and followed**	0	0	1	100	1	100	0	0	2	67	0	0	1	100
	**Procedures exist but not followed**	1	100	0	0	0	0	0	0	0	0	1	100	0	0
	**No procedures**	0	0	0	0	0	0	24	100	1	33	0	0	0	0
**Finance and Administration**															
**Q34: Have you previously acquired grants/funding for research?**	**Yes**	0	0	0	0	0	0	0	0	2	67	0	0	0	0
	**No**	1	100	1	100	1	100	24	100	1	33	1	100	1	100
**Q35: How much time can admin staff allocate to research support (average hours per staff)?**	**<4 hours/week per staff**	0	0	1	100	1	100	0	0	3	100	1	100	1	100
	**4-8 hours/week per staff**	0	0	0	0	0	0	24	100	0	0	0	0	0	0
	**8-12 hours/week per staff**	0	0	0	0	0	0	0	0	0	0	0	0	0	0
	**12-16 hours/week per staff**	0	0	0	0	0	0	0	0	0	0	0	0	0	0
	**>16 hours/week per staff**	1	100	0	0	0	0	0	0	0	0	0	0	0	0
**Collaboration and Networking**															
**Q36: Does the clinic participate in diabetes research networks?**	**National + international networks**	1	100	1	100	1	100	0	0	0	0	1	100	0	0
	**National networks only**	0	0	0	0	0	0	24	100	0	0	0	0	0	0
	**Does not participate**	0	0	0	0	0	0	0	0	3	100	0	0	1	100
